# Optimally Miscible Polymer Bulk-Heterojunction-Particles for Nonsurfactant Photocatalytic Hydrogen Evolution

**DOI:** 10.1021/jacs.4c13856

**Published:** 2024-12-20

**Authors:** Wei-Cheng Lin, Yu-En Sun, Ying-Rang Zhuang, Tse-Fu Huang, Kuei-Jhong Lin, Mohamed M. Elsenety, Jui-Chen Yen, Hung-Kai Hsu, Bo-Han Chen, Chen-Yu Chang, Je-Wei Chang, Hsin-Ni Huang, Bing-Heng Li, Siriporn Jungsuttiwong, Toton Haldar, Shin-Huei Wang, Wan-Chi Lin, Tien-Lin Wu, Chin-Wen Chen, Chi-Hua Yu, An-Chung Su, Kun-Han Lin, U-Ser Jeng, Shang-Da Yang, Ho-Hsiu Chou

**Affiliations:** † Department of Chemical Engineering, National Tsing Hua University, Hsinchu 300044, Taiwan; ‡ Department of Chemistry, Faculty of Science, Al-Azhar University, Nasr City, Cairo 11884, Egypt; § Institute of Photonics Technologies & Department of Electrical Engineering, National Tsing Hua University, Hsinchu 300044, Taiwan; ∥ National Synchrotron Radiation Research Center, Hsinchu 30076, Taiwan; ⊥ Department of Chemistry and Center of Excellence for Innovation in Chemistry, Faculty of Science, Ubon Ratchathani University, Ubon Ratchathani 34190, Thailand; # Department of Engineering Science, National Cheng Kung University, Tainan 701401, Taiwan; ∇ Department of Molecular Science and Engineering, National Taipei University of Technology, Taipei 106344, Taiwan; ○ Department of Chemistry, National Tsing Hua University, Hsinchu 300044, Taiwan; ◆ Center for Photonics Research, National Tsing Hua University, Hsinchu 300044, Taiwan; ¶ College of Semiconductor Research, National Tsing Hua University, Hsinchu 300044, Taiwan

## Abstract

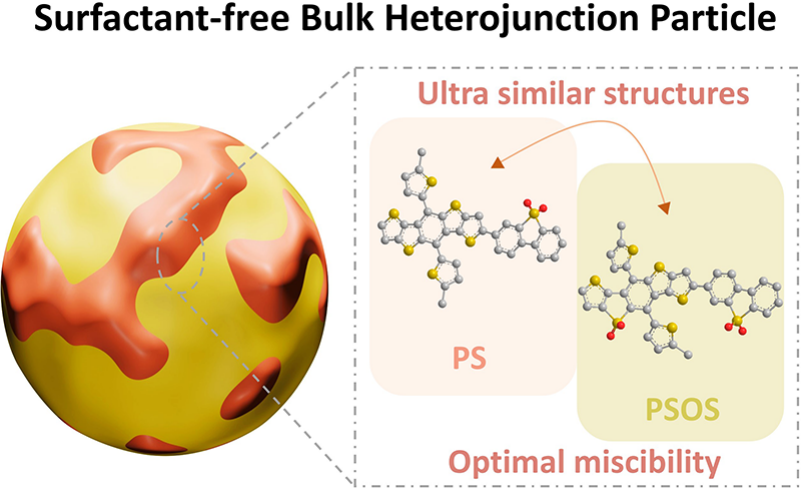

Mini-emulsion and nanoprecipitation techniques relied on large amounts of surfactants, and unresolved miscibility issues of heterojunction materials limited their efficiency and applicability in the past. Through our molecular design and developed surfactant-free precipitation method, we successfully fabricated the best miscible bulk-heterojunction-particles (BHJP) ever achieved, using donor (**PS**) and acceptor (**PSOS**) polymers. The structural similarity ensures optimal miscibility, as supported by the interaction parameter of the **PS**/**PSOS** blend is positioned very close to the binodal curve. Experimental studies and molecular dynamics simulations further revealed that surfactants hinder electron output sites and reduce the concentration of sacrificial agents at the interface, slowing polaron formation. Multiscale experiments verified that these BHJP, approximately 12 nm in diameter, further form cross-linked fractal networks of several hundred nanometers. Transient absorption spectroscopy showed that BHJP facilitates polaron formation and electron transfer. Our BHJP demonstrated a superior hydrogen evolution rate (HER) compared to traditional methods. The most active BHJP achieved an HER of 251.2 mmol h^–1^ g^–1^ and an apparent quantum yield of 26.2% at 500 nm. This work not only introduces a practical method for preparing BHJP but also offers a new direction for the development of heterojunction materials.

## 1 Introduction

A paradigm shift from fossil fuels to carbon-neutral energy sources is imperative to address the challenges of climate change. While solar energy is recognized as the most abundant and sustainable renewable resource, its intrinsic intermittency hinders consistently meeting energy demands;^1^ consequently, the capture and storage of solar energy within the chemical bonds of fuel become imperative. Hydrogen, synthesized through solar energy via water splitting, emerges as a highly promising solar fuel.^2^ Specifically, organic semiconductors, valued for attributes such as low-temperature processing, cost-effectiveness, easily modifiable molecular structure, and tunable bandgaps, are selected as photocatalysts to achieve exceptional hydrogen evolution.^3,4^ Recently, the array of organic semiconductors employed for hydrogen evolution includes linear conjugated polymers,^5−7^ conjugated microporous polymers,^8,9^ hydrophilic conjugated polymers,^10,11^ covalent organic frameworks,^12−14^ and photovoltaic catalysts.^15,16^

However, single organic photocatalysts typically face challenges such as high exciton binding energies and limited exciton diffusion lengths, which lead to exciton recombination and inefficient charge generation.^17^ Most research has broadly adopted strategies involving nanosized particle fabrication with heterojunction interfaces to overcome this limitation. This approach blends photoactive polymers/molecules as donor and acceptor components within the same nanoparticle (NP), forming internal donor–acceptor (D-A) heterojunction. Such interface designs can facilitate rapid exciton separation, inhibit recombination, and prevent energy loss.^18−21^

Currently, the primary techniques for preparing D–A heterojunction particles are mini-emulsion and nanoprecipitation methods.^22,23^ However, these techniques have inherent limitations. The preparation processes are often time-consuming and complex, involving multiple steps for solvent removal and repetitive precipitation. Additionally, these methods face challenges in increasing the concentration of photocatalysts. Maintaining a uniform reaction system is crucial, and excessively high polymer concentrations can cause phase separation or aggregation, impacting particle size and structural control. On the other hand, the inclusion of surfactants becomes imperative to stabilize particle formation and dispersion. Nevertheless, using surfactants poses a potential risk of impeding the light absorption capacity of photocatalyst and interfering with the electron transfer process. Furthermore, surfactants significantly increase the overall cost of the preparation process.

Beyond these challenges, miscibility issues in heterojunction materials impact various organic electronic technologies, including organic photovoltaics, photodetectors, and organic photocatalytic systems.^24^ Low D–A miscibility (hypo-miscibility) can lead to performance degradation due to overpurification of mixed domains,^25^ while high miscibility (hyper-miscibility) results in poor phase separation and reduced performance.^26^ Despite years of research, no breakthroughs have been made in achieving optimal miscibility through molecular engineering of donor and acceptor materials.

**Figure 1:**
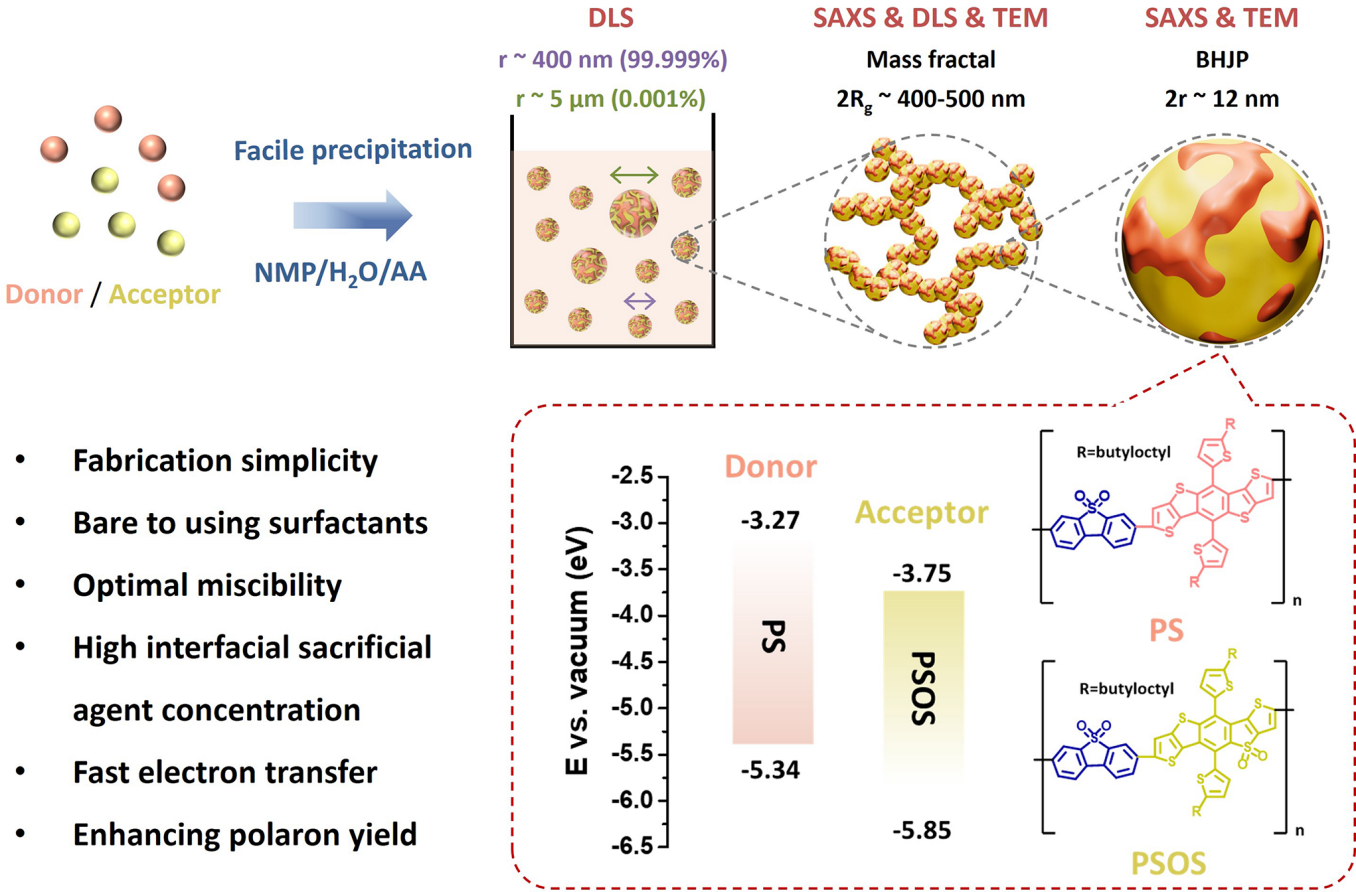
Schematic illustration depicting the preparation of **PS** and **PSOS** BHJP using a facile precipitation method, along with the molecular structures and energy levels of the donor polymer **PS** and the acceptor polymer **PSOS**.

In this work, we present a facile precipitation method for producing thermodynamically stable bulk**-**heterojunction**-**particles (BHJP) using donor Poly(5,10-bis(5-(2-butylocxyl)-2-thiophenyl) dithieno[2,3-d:2′,3′-d’]benzo[1,2-b:4,5-b’]dithiophene-*alt*-dibenzo[b,d]thiophene 5,5-dioxide) (**PS**) and acceptor Poly(5,10-bis(5-(2-butylocxyl)-2-thiophenyl) dithieno[2,3-d:2′,3′-d’]benzo[1,2-b:4,5-b’]dithiophene 1,1-dioxide-*alt*-dibenzo[b,d]thiophene 5,5-dioxide) (**PSOS**). **PS** and **PSOS** possess similar structures, with the primary distinction lying in the presence of an additional sulfone functional group in the **PSOS** structure. This leads to a 0.5 eV downshift in both HOMO and LUMO energy levels, resulting in a favorable type II energy level offset. The structural similarity between these polymers ensures optimal miscibility, supported by the interaction parameter (χ) of the **PS**/**PSOS** blend, which is close to the binodal curve. To the best of our knowledge, this design strategy using such structurally similar donor and acceptor materials is unprecedented. Small angle X-ray scattering (SAXS) and dynamic light scattering (DLS) analyses reveal the structure and morphology of these BHJP. More importantly, our comparative analysis with established techniques, such as mini-emulsion and nanoprecipitation, highlights the advantages of this new method, including its versatility, simplicity, cost-effectiveness, surfactant-free nature, and high efficiency. Transient absorption spectroscopy demonstrated that prepared BHJP facilitates the formation of electron polarons and accelerates electron transfer due to the lack of surfactants as confirmed by molecular dynamic simulation (Figure 1).

## 2 Results and Discussion

### 2.1 Compatibility and BHJP Morphology Characterization

**PS** and **PSOS** were prepared through the Pd-catalyzed Suzuki–Miyaura coupling method. The incorporation of long alkyl side chains significantly enhanced their solubility in common organic solvents like chloroform, 1-methyl-2-pyrrolidone (NMP), and tetrahydrofuran (THF), facilitating the preparation of heterojunction particles. The HOMO/LUMO energy levels for **PS** and **PSOS** are −5.34/–3.27 eV and −5.85/–3.75 eV, respectively, indicating a favorable type II energy level alignment conducive to efficient exciton dissociation and charge transfer. These two polymers are the same as those used in our previous studies, and measurements of their fundamental properties and results are provided in the Supporting Information.^27^

**Figure 2:**
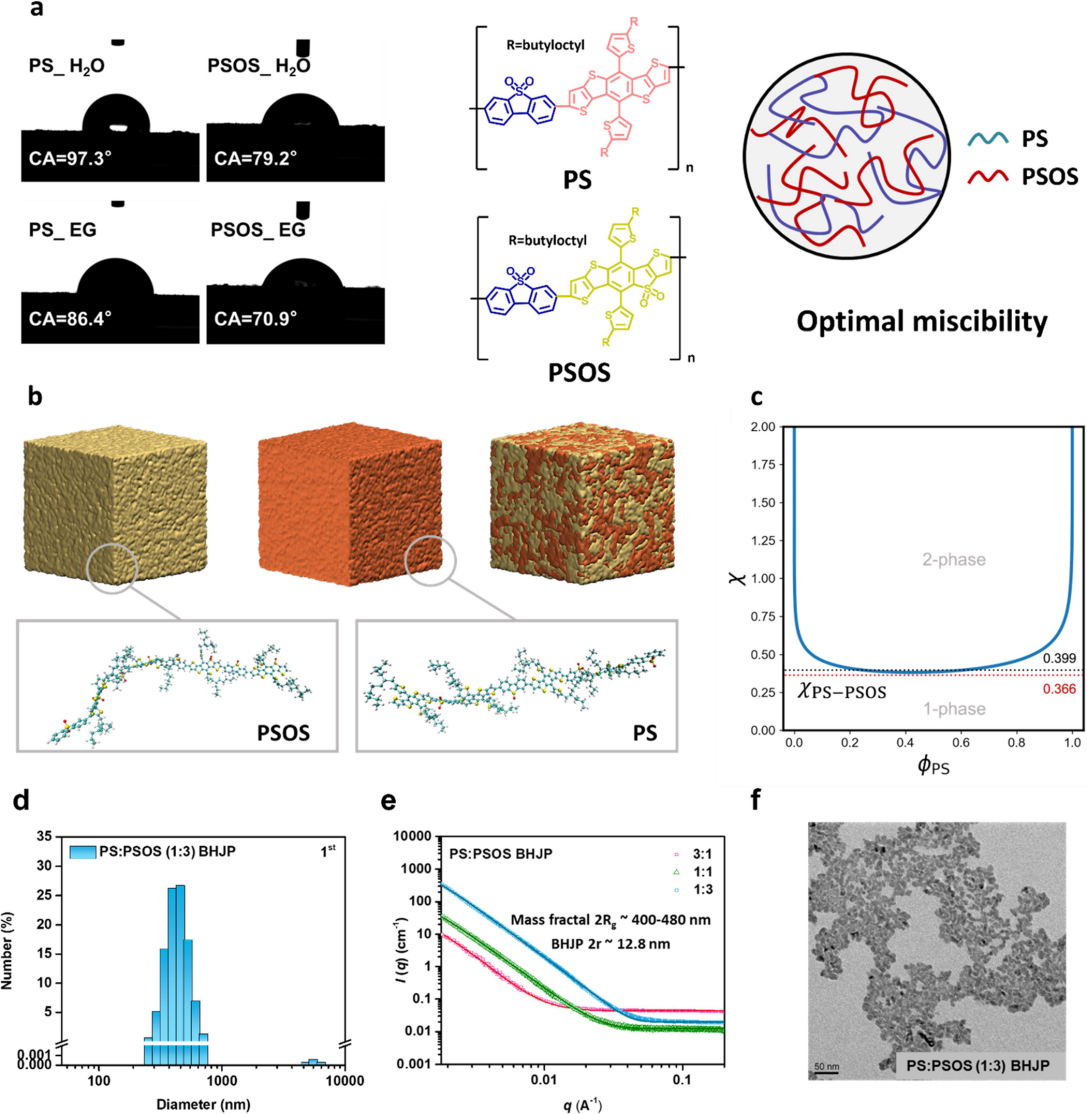
(a) Contact angle with water and ethylene glycol, chemical structures, and compatibility of **PS** and **PSOS**, (b) solid-state structures with varying proportions of polymer chains (tetramer): 1000 **PS** chains (orange), 1000 **PSOS** chains (yellow), and a polymer blend of 500 **PS** and 500 **PSOS** chains, (c) the phase diagram of the **PS**/**PSOS** blend constructed using Flory–Huggins theory. Degree of polymerization of **PS** and **PSOS** are 9 and 6, respectively, (d) DLS data for **PS**:**PSOS** (1:3) BHJP, (e) SAXS data measured for the solutions of **PS**/**PSOS** prepared in different mixing ratios as indicated (same sample wt % concentration). Data are fitted (solid curves) using the fractal model described by the primary particles of radius *r*_d_, fractal dimension *D*_f_, and cutoff length ξ, and (f) TEM image of **PS**:**PSOS** (1:3) BHJP. We note that these DLS and SAXS data are of reproducibility.

To demonstrate the excellent compatibility from structural similarity, contact angles (CAs) of pure **PS** and **PSOS** films were measured using water and ethylene glycol (EG) drops. As a control, Poly[(2,6-(4,8-bis(5-(2-ethylhexyl-3-fluoro)thiophen-2-yl)-benzo[1,2-b:4,5-b′]dithiophene))-*alt*-(5,5-(1′,3′-di-2-thienyl-5′,7′-bis(2-ethylhexyl)benzo[1′,2′-c:4′,5′-c′]dithiophene-4,8-dione)] (**PM6**) and 2,2′-((2Z,2′Z)-((12,13-bis(2-ethylhexyl)-3,9-diundecyl-12,13-dihydro-[1,2,5]thiadiazolo[3,4-*e*]thieno[2″,3″:4′,5′]thieno[2′,3′:4,5]pyrrolo[3,2-g]thieno[2′,3′:4,5]thieno[3,2-*b*]indole-2,10-diyl)bis(methaneylylidene))bis(5,6-difluoro-3-oxo-2,3-dihydro-1H-indene-2,1-diylidene))dimalononitrile (**Y6**) were also evaluated. **PM6** and **Y6** were selected as the control group because they are well-established benchmark D–A heterojunction materials in organic photovoltaics and photocatalysis.^21,28^Figures 2a, S1 and Table S1 show the CAs and calculated surface energy (γ) values: 20.6, 22.8, 27.2, and 34.7 mN m^–1^, respectively.^29^ These results highlight the close surface energy of **PS** and **PSOS**. According to Flory–Huggins theory, the interaction parameter (χ) is a dimensionless quantity that distinguishes the interaction energy differences between polymer–polymer and polymer-small molecule blends. The χ value between **PS** and **PSOS** is 0.054 K, where K is a proportional constant (see Supporting Information). This value is significantly lower than the 0.448 K for **PM6**-**Y6**, indicating high interfacial compatibility and a low enthalpy of mixing between the donor and acceptor, suppressing phase separation and promoting intermixed domains with large D–A interfaces in the blends.^30,31^

To estimate the χ between **PS** and **PSOS**, we conducted molecular dynamics (MD) simulations using tetramers as representatives for the polymers (Figure 2b). Details of the simulation protocol are provided in the SI and Figure S2. To ensure the accuracy of our simulations, we first calculated the solubility parameters for **PS** and **PSOS**, obtaining values of 16.985 and 18.016 MPa^1/2^, respectively. These results closely align with experimental data presented in Table S2, affirming the reliability of our force-field parameters and simulation methodology. Using the validated approach, we calculated the χ for the **PS**/**PSOS** blend using two different methods.^32^ Without considering the mixing potential energy, the χ value was constant at 0.366. Additionally, when the mixing potential energy was included, the χ values were 0.399 for the 1:1 blend and 0.415 for the 1:3 blend. The slight increase in χ for the 1:3 blend reflects minimal variation, confirming consistent miscibility between **PS** and **PSOS** across different ratios. The methods and detailed results are provided in the Supporting Information and summarized in Table S3. Figure 2c illustrates the phase diagram of the **PS**/**PSOS** blend, constructed using Flory–Huggins theory.^25^ Notably, the χ of the **PS**/**PSOS** blend is positioned very close to the binodal curve, indicating optimal miscibility.^33^ This proximity suggests that the **PS**/**PSOS** BHJP is thermodynamically stable and less prone to performance degradation due to excessive purification of the mixed domains.^34^ Further exploration of the stability and long-term photocatalytic activity of the **PS**/**PSOS** BHJP could be crucial for designing stable photocatalysts.

Next, we investigated the structure and morphology of particles prepared by the facile precipitation method. DLS measurements revealed a bimodal particle size distribution, with smaller particles ranging from 200 to 700 nm in diameter constituting the majority, while larger aggregates of approximately 5 μm accounted for only 0.001–0.01% of the total (Figures 2d and S3). To further study the structure of BHJP, we used SAXS and Ultrasmall-Angle X-ray Scattering (USAXS) measurements under identical conditions. Figure 2e displays the three intensity profiles *I*(*q*) as a function of the scatter vector *q* for the three distinct sample solutions with **PS** and **PSOS** ratios of 3:1, 1:1, and 1:3. The low-*q* region of the data shows a power-law behavior represented by *I*(*q*) ∝ *q*^–α^. The fitted α values of 2.86, 2.74, and 2.74 (±0.01) suggest the presence of mass fractal aggregates with corresponding mass fractal dimensions. Additionally, the data were analyzed using a fractal model comprising polymer particles with a radius (*r*) of around 6 nm and a radius of gyration (*R*_g_) in the range of 200–240 nm, determined from the fitted fractal cutoff length and fractal dimension. Detailed results of the fitting process are presented in Figures S4 and S5, with the corresponding structural parameters summarized in Tables S4 and S5. To explore the potential interaction between the donor polymer **PS** and the acceptor polymer **PSOS**, we also measured the SAXS of a mixture of both pristine particles. The results show a significantly higher scattering intensity for the **PS**:**PSOS** (1:3) BHJP compared to the **PS**:**PSOS** (1:3) mixed pristine particles, suggesting the formation of larger particles from the complexation of **PS** and **PSOS** (Figure S6). The BHJP formation mechanism is further discussed in terms of polymer chain arrangements, as shown in Figure S7. To provide more intuitive support for the structural analysis, we characterized the morphology and particle size of the **PS**:**PSOS** (1:3) BHJP via transmission electron microscopy (TEM). As shown in Figure 2f, the TEM image reveals that the BHJP exhibits a mass fractal-like morphology with aggregated nanoparticles forming a branched, interconnected network. This structural arrangement aligns well with the particle size distribution and morphology measured by SAXS and DLS, providing complementary evidence for the structural analysis. The TEM images of the **PS**:**PSOS** (1:3) BHJP at different magnifications are provided in Figure S8. The surface area and porosity of the **PS**:**PSOS** (1:3) BHJP in the powder state were further characterized using Brunauer–Emmett–Teller (BET) measurements, revealing a surface area of 11.75 m^2^ g^–1^ and a total pore volume of 0.077 cm^3^ g^–1^. While these measurements offer insights into the surface properties of materials, they may not directly represent the surface area under photocatalytic reaction conditions (Figure S9).

### 2.2 Photocatalytic H_2_ Evolution of BHJP Prepared via Facile Precipitation Method

**Figure 3:**
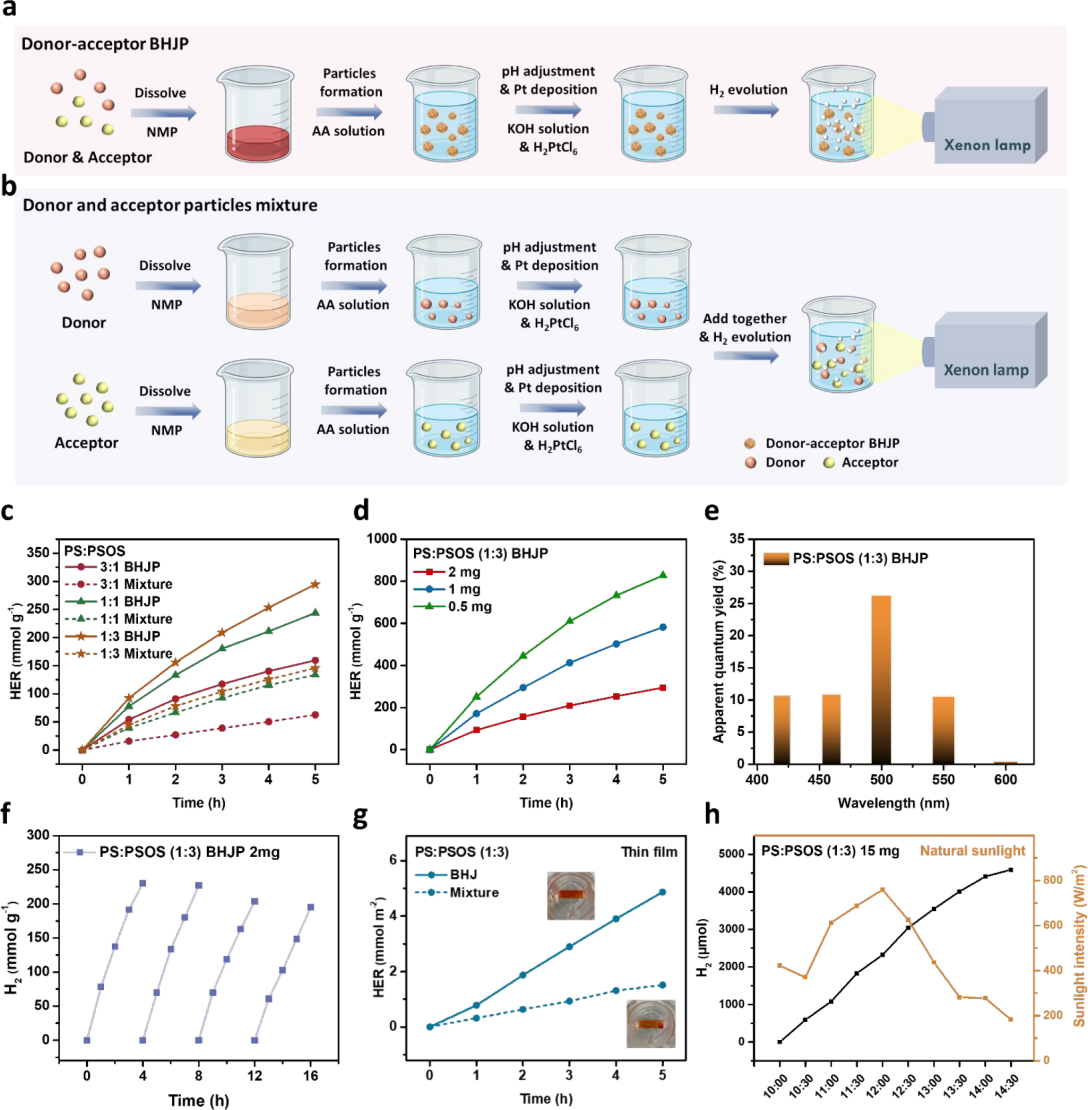
(a) Schematic illustration of BHJP preparation using the facile precipitation method, (b) Schematic illustration of pristine particle mixture preparation, (c) HER over time for **PS**/**PSOS** BHJP and pristine particle mixtures with various **PS**/**PSOS** ratios. Conditions: 2 mg photocatalyst in 10 mL water/NMP (9:1 v/v), 1 M AA, and 3 wt % H_2_PtCl_6_, (d) HER of **PS**:**PSOS** (1:3) BHJP at different photocatalyst dosages (2/1/0.5 mg). Conditions: 10 mL water/NMP (9:1 v/v), 1 M AA, and 3 wt % H_2_PtCl_6_, (e) AQY of **PS**:**PSOS** (1:3) BHJP at 420, 460, 500, 550, and 600 nm. Conditions: 2 mg photocatalyst in 10 mL water/NMP (9:1 v/v), 1 M AA, and 3 wt % H_2_PtCl_6_, (f) photocatalytic cycling stability test of **PS**:**PSOS** (1:3) BHJP. Conditions: 2 mg photocatalyst in 10 mL water/NMP (9:1 v/v), 1 M AA, and 3 wt % H_2_PtCl_6_, (g) HER comparison between **PS**:**PSOS** (1:3) BHJ thin film and mixture thin film. Conditions: 2 mg photocatalyst in 10 mL water/NMP (9:1 v/v), 1 M AA, and 3 wt % H_2_PtCl_6_, and (h) HER of **PS**:**PSOS** (1:3) BHJP under natural sunlight, with solar irradiation intensity tracked throughout the day. Conditions: 15 mg photocatalyst in 300 mL water/NMP (9:1 v/v), 1 M AA, and 3 wt % H_2_PtCl_6_.

The synthesis of BHJP, using a novel and facile precipitation method, is detailed in the experimental section and shown in Figure 3a. A control group was established to assess the effectiveness of this approach in forming D–A heterojunctions by separately preparing and mixing acceptor and donor particles, as shown in Figure 3b. Both methods used identical solution conditions, differing only in the formation of BHJP versus mixing pristine particles. Hydrogen evolution tests were performed at ambient temperature under visible light irradiation (λ = 380–780 nm) with a cutoff filter and an irradiance intensity of 1000 W m^–2^. The reaction medium consisted of a 10 mL NMP/water mixture (1:9 v/v) with ascorbic acid (AA) as a sacrificial agent and 3 wt % H_2_PtCl_6_ as a cocatalyst. Efficiency analysis of BHJP with varying **PS**/**PSOS** mass ratios showed a significant increase in activity compared to the pristine particle mixture (Figure 3c), attributed to superior polaron charge generation in the BHJP, facilitated by the D–A heterojunction. Moreover, the BHJP with an optimal **PS** to **PSOS** mass ratio of 1:3 demonstrated superior efficiency compared to the 1:1 and 3:1 ratios (Figure 3c), which is attributed to its exceptional light absorption capability (Figure S10). The superior performance of the **PS**:**PSOS** (1:3) BHJP can also be attributed to its structural characteristics. The SAXS measurements reveal a relatively large *R*_g_ value for **PS**:**PSOS** (1:3) BHJP, suggesting a lower packing density of the mass fractal aggregates compared to **PS**:**PSOS** (3:1) BHJP and **PS**:**PSOS** (1:1) BHJP (Table S4) This reduced packing density provides more permeation space within the polymer mass fractal aggregates, facilitating greater water accessibility and enhancing photocatalytic efficiency.^10,35^ Further fine-tuning of the polymer dosage to 0.5 mg elevated the average hydrogen evolution rate of the **PS**:**PSOS** (1:3) BHJP to a maximum of 251.2 mmol h^–1^ g^–1^, surpassing the majority of organic conjugated polymer materials (Figures 3d, S11 and Table S6). The reduced polymer loading effectively suppressed particle aggregation, minimizing the formation of larger particles and improving the dispersion of the polymer in the reaction medium (Figure S12). This improved dispersion facilitated superior light absorption and transmission, thereby maximizing the utilization efficiency of active sites within the BHJP. The photocatalytic hydrogen evolution performance of **PS**:**PSOS** (1:3) BHJP was evaluated at different pH levels, as shown in the Figure S13. When AA was used as the sacrificial agent, the reaction under acidic conditions (pH 4) achieved the highest hydrogen evolution rate. In contrast, at neutral (pH 7) and alkaline (pH 11) conditions, the HER significantly decreased due to the instability of AA, which oxidizes into dehydroascorbic acid and other products with insufficient electron-donating capability.

However, the hydrogen evolution rate (HER) based on the total mass of the catalyst may lead to a flawed analysis of the efficiency of photocatalyst.^36,37^ Therefore, we evaluated the apparent quantum yield (AQY) by comparing the incident photonic numbers with the electrons utilized for hydrogen evolution to explore the wavelength-dependent photocatalytic activity. Notably, the **PS**:**PSOS** (1:3) BHJP achieved a photon-to-hydrogen conversion AQY of 26.2% at 500 nm (Figure 3e). We employed fluorescence microscopy to observe the photocatalytic hydrogen evolution process, chosen for its ability to image particles in an environment resembling their native state during hydrogen evolution. Supplementary Videos 1 and 2 illustrate the photocatalytic hydrogen evolution process of **PS**:**PSOS** (1:3) BHJP and a mixture of both pristine particles, respectively. The videos showcase abundant bubbles under light irradiation, indicating hydrogen generation. Additionally, **PS**:**PSOS** (1:3) BHJP demonstrates a notably accelerated rate of hydrogen bubble generation compared to the mixture of both pristine particles. The photostability of **PS**/**PSOS** BHJP in a photocatalytic reaction was assessed through a continuous four-cycle experiment in a NMP/water/AA mixed solution. As shown in Figure 3f, after 16 h of continuous hydrogen evolution (4 cycles), the HER of 2 mg **PS**/**PSOS** (1:3) BHJP retained approximately 85% of its initial activity, while 0.5 mg **PS**/**PSOS** (1:3) BHJP maintained 78% after the fourth cycle (Figure S14). Nuclear magnetic resonance analysis, X-ray photoelectron spectroscopy (XPS), Fourier-transform infrared spectroscopy (FTIR) revealed no significant differences between the recovered **PS**/**PSOS** BHJP and the as-prepared samples, confirming the structural stability of the polymer without signs of degradation (Figures S15–S18). The observed efficiency reduction is likely attributed to self-inhibition caused by AA and its oxidation product, dehydroascorbic acid.

To assess the applicability of our method to thin films, we prepared a **PS**/**PSOS** heterojunction thin film via a single drop-casting cycle on a UV-ozone-treated glass substrate (active area: 4.5 cm^2^). UV-ozone treatment improved polymer adhesion to the glass substrate, effectively preventing delamination during light irradiation. Photocatalytic hydrogen evolution tests were conducted with the thin film submerged in a NMP/water/AA mixture. As shown in Figure 3g, the **PS**/**PSOS** heterojunction thin film achieved a HER of 4.9 mmol m^–2^ h^–1^. For comparison, a control experiment using separate **PS** and **PSOS** thin films (1:3 ratio) on the same active area (4.5 cm^2^) yielded a HER of only 1.5 mmol m^–2^ h^–1^ (Figure S19), more than 3-fold lower than the heterojunction thin film. These results confirm that our D–A heterojunction material design strategy is effective for both particle and thin film systems. To further demonstrate the versatility and applicability of our design approach, we extended this method to other polymer-based systems. Specifically, we used the polymers **PITS** and **PITDSOS**, developed in our previous research,^27^ were employed to prepare **PITS**:**PITDSOS** (1:3) BHJP via the facile precipitation method and compare its photocatalytic activity with **PITS**:**PITDSOS** (1:3) pristine particle mixture. As shown in Figure S20, the **PITS**:**PITDSOS** (1:3) BHJP exhibited a more than 2-fold enhancement in HER compared to the pristine particle mixture, underscoring the effectiveness of the heterojunction structure formed through our method. This improvement demonstrates the adaptability of our strategy to various thiophene-based multifused ladder-type heteroarenes, highlighting its broad potential for diverse polymer-based photocatalysts. To evaluate the scalability and performance of **PS**:**PSOS** (1:3) BHJP under real-world conditions, we conducted photocatalytic hydrogen evolution experiments using natural sunlight. A 30-fold scaled-up system was implemented in a custom-designed reactor with a diameter of 20 cm and a height of 3.5 cm (Figure S21). The reaction solution consisted of 15 mg of **PS**:**PSOS** (1:3) in 30 mL of NMP, 270 mL of deionized water, 3 wt % H_2_PtCl_6_, and 1 M AA, adjusted to pH 4. HER measurements were conducted at 30 min intervals between 10:00 AM and 2:00 PM under an average natural sunlight intensity of 400–500 W m^–2^. Over 4 h, the total hydrogen evolution reached 4407 μmol (Figure 3h). In comparison to a smaller-scale experiment using the same concentration (0.5 mg of **PS**:**PSOS** (1:3) in 1 mL of NMP, 9 mL of deionized water, 3 wt % H_2_PtCl_6_, and 1 M AA), the total hydrogen production increased by a factor of 12 rather than the expected 30-fold. This discrepancy is attributed to the lower sunlight intensity in the natural sunlight setup, which was approximately 2–2.5 times weaker than the controlled laboratory conditions (Figure S22). These findings highlight the scalability of **PS**:**PSOS** (1:3) BHJP for hydrogen production under real-world conditions, while also emphasizing the influence of environmental factors, such as sunlight intensity, on performance.

**Figure 4:**
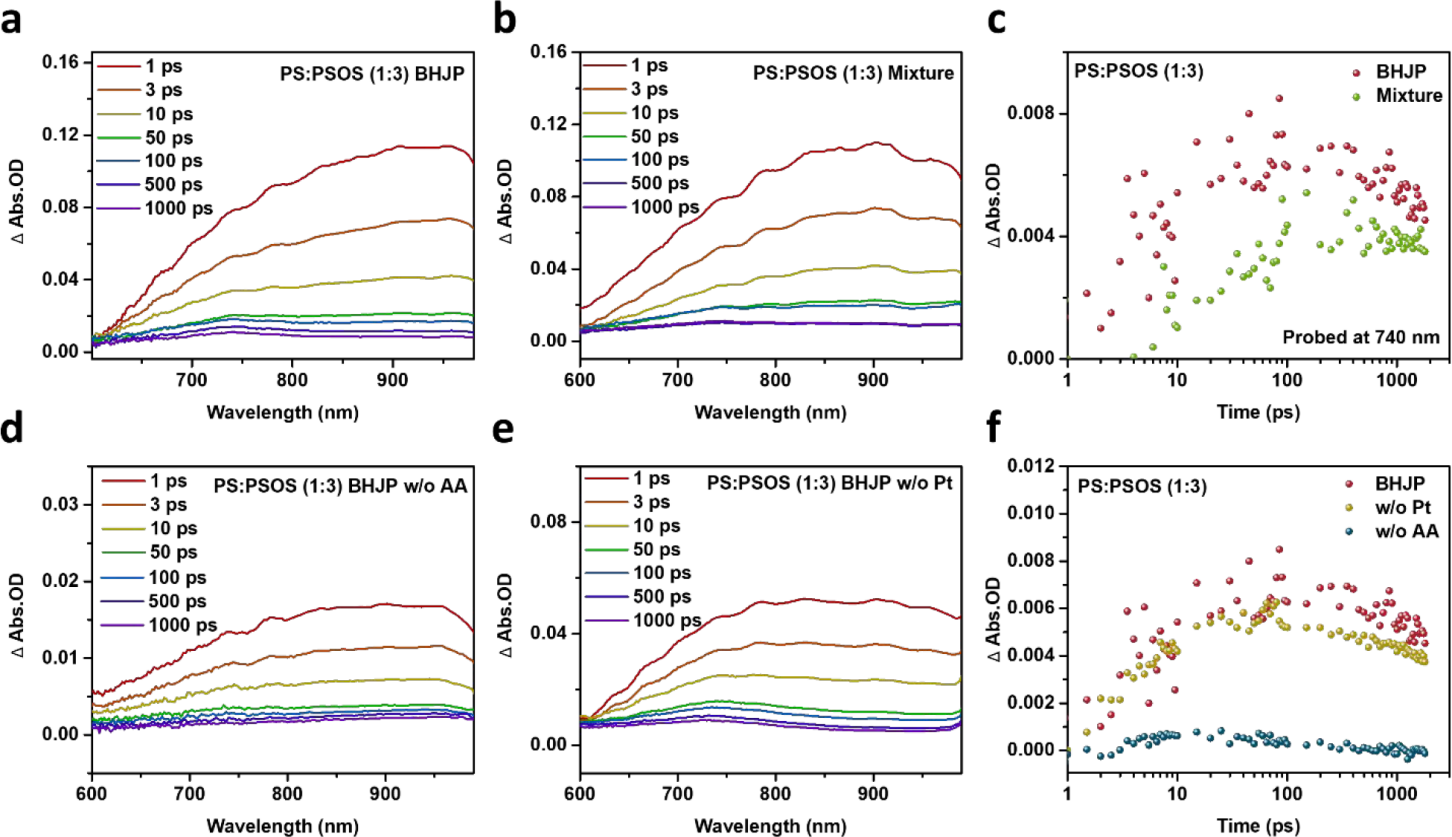
(a) TAS of **PS**:**PSOS** (1:3) BHJP, (b) TAS of the **PS**:**PSOS** (1:3) pristine particle mixture at various time delays following excitation at 480 nm, (c) deconvoluted transient absorption kinetics of **PS**:**PSOS** (1:3) BHJP and **PS**:**PSOS** (1:3) pristine particle mixture polarons obtained through analysis of the data shown in (a) and (b), TAS of **PS**:**PSOS** (1:3) BHJP at different time delays following excitation at 480 nm (d) without AA, (e) without Pt and (f) deconvoluted transient absorption kinetics to polarons obtained through analysis of the data shown in (a), (d), and (e).

We used femtosecond-to-nanosecond transient absorption spectroscopy (TAS) to investigate charge carrier dynamics in the photocatalysts. Figure 4a,b delineate the TAS results at delay times spanning from 1 ps (ps) to 1 ns (ns) for two types of photocatalysts, **PS**:**PSOS** (1:3) BHJP and **PS**:**PSOS** (1:3) mixed pristine particles, both subjected to 480 nm excitation in the hydrogen evolution solution. In all polymers, a positive signal beyond 600 nm corresponds to photoinduced absorption (PIA), indicating singlet exciton absorption. Furthermore, we observed an additional signal with an extended half-life in the wavelength range of 700–800 nm was observed within the PIA spectrum. This additional signal is attributed to conjugated polymers that, upon light excitation and electron acquisition from sacrificial agents, develop partial charges and generate electron polarons.^5,38,39^ We conducted a detailed deconvolution of the PIA signals into exciton and polaron components on ultrafast time scales at 740 nm. As illustrated in Figure 4c, these findings regarding the polaron signal underscore that BHJP exhibits a higher population of electron polarons than mixed pristine particles. The increased charge generation in BHJP contributes to a higher yield of catalytically active charges, thus enhancing the observed HER compared to their mixed pristine particle counterparts. To further identify the contributors to the PIA signal, we performed a comparative TAS analysis on BHJP in two conditions: in hydrogen evolution solution without a Pt cocatalyst and in hydrogen evolution solution without AA (Figure 4d,e). Figure 4f clearly shows that the absence of the sacrificial agent AA significantly diminishes the electron polaron signal. This observation underscores the critical role of hole scavenger AA in generating long-lived electron polarons in this system, consistent with previous literature.^21,38^ Consequently, optimizing the electron transfer between the polymer and the hole scavenger is essential for enhancing the population of electron polarons.

### 2.3 Effect of Surfactants Addition on the Photocatalytic H_2_ Evolution

**Figure 5:**
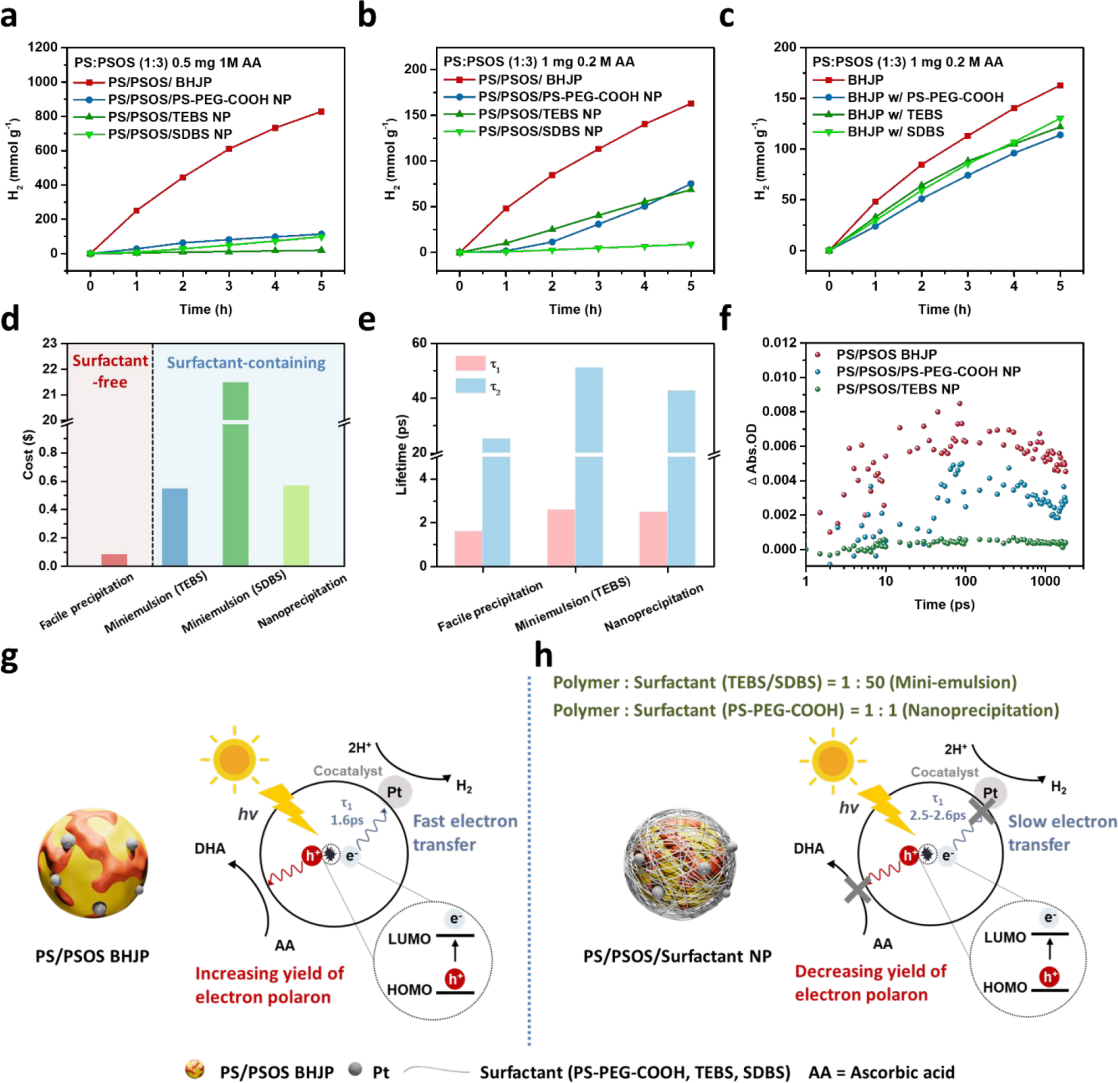
(a) HER for **PS/PSOS** BHJP, **PS/PSOS/PS–PEG-COOH** NP, **PS/PSOS/TEBS** NP, and **PS/PSOS/SDBS** NP. Conditions: 0.5 mg photocatalyst, 1 M AA, and 3 wt % H_2_PtCl_6_, (b) HER for **PS/PSOS** BHJP, **PS/PSOS/PS–PEG-COOH** NP, **PS/PSOS/TEBS** NP, and **PS/PSOS/SDBS** NP. Conditions: 1 mg photocatalyst, 0.2 M AA, and 3 wt % H_2_PtCl_6_, (c) HER for **PS**: **PSOS** (1:3) BHJP with and without added surfactants, including TEBS, SDBS, and PS–PEG-COOH. Conditions: 1 mg photocatalyst, 0.2 M AA, and 3 wt % H_2_PtCl_6_, (d) the cost of various methods employed to prepare heterojunction particles, (e) shorter lifetime components (τ_1_ and τ_2_) of the PIA signal fitting curve for **PS**/**PSOS** heterojunction particles prepared by different methods at 901 nm, (f) deconvoluted transient absorption kinetics to polarons obtained through analysis of the data shown in Figures S26 and 4a, (g) schematic diagram of the **PS**/**PSOS** BHJP prepared by facile precipitation method, and (h) schematic diagram of the **PS**/**PSOS/**surfactant NP prepared by nanoprecipitation and mini-emulsion.

Additionally, we compared BHJP synthesized via a facile precipitation method with heterojunction particles prepared using mini-emulsion and nanoprecipitation techniques. The heterojunction structure of **PS** and **PSOS** was synthesized using the mini-emulsion method with surfactants such as sodium 2-(3-thienyl)ethyloxybutylsulfonate (TEBS) and sodium dodecyl benzenesulfonate (SDBS) (Figure S23c), and the nanoprecipitation method with polystyrene graft ethylene oxide functionalized with carboxylic acid (PS–PEG-COOH) (Figure S23d), resulting in three distinct heterojunction NP: **PS**/**PSOS/TEBS** NP, **PS**/**PSOS/SDBS** NP, and **PS**/**PSOS/PS–PEG-COOH** NP. These surfactants are representative of those commonly employed in traditional synthetic methods like nanoprecipitation and mini-emulsion, where they stabilize nanoparticles and enhance dispersion stability.^19,21^ In the mini-emulsion method, the surfactant-to-polymer weight ratio can be as high as 50:1, whereas in the nanoprecipitation method, the ratio is typically equivalent between surfactant and polymer. DLS measurements revealed that the hydrodynamic diameters of **PS**/**PSOS/TEBS** NP, **PS**/**PSOS/SDBS** NP, and **PS**/**PSOS/PS–PEG-COOH** NP ranged from 30 to 120 nm (Figure S24), which were slightly smaller than those of **PS**/**PSOS** BHJP synthesized via the facile precipitation method. To further investigate the photocatalytic hydrogen evolution, various particle formation methods were evaluated under standardized conditions: 0.5 mg photocatalyst, 1 M AA, and 3 wt % H_2_PtCl_6_. As shown in Figure 5a, the HER of **PS**:**PSOS** (1:3) BHJP significantly outperforms that of heterojunction nanoparticles prepared by nanoprecipitation and mini-emulsion approaches. To provide a more detailed comparison, hydrogen evolution tests were conducted under conditions commonly used for heterojunction particles: 1 mg photocatalyst, 0.2 M AA, and 3 wt % H_2_PtCl_6_. As presented in Figure 5b, **PS**:**PSOS** (1:3) BHJP achieved an HER of 163.9 mmol g^–1^ 5 h^–1^, surpassing **PS**:**PSOS** /PS–PEG-COOH NP (75.3 mmol g^–1^ 5 h^–1^), **PS**:**PSOS** /TEBS NP (68.6 mmol g^–1^ 5 h^–1^), and **PS**:**PSOS** /SDBS NP (9.0 mmol g^–1^ 5 h^–1^). These findings demonstrate that the **PS**:**PSOS** BHJP exhibits 218–1821% higher efficiency compared to **PS**:**PSOS** /surfactant nanoparticles, emphasizing the versatility and superiority of the facile precipitation method. Additionally, to clarify the benefits of fabricating heterojunction particles without additional surfactants, we examined the effects of added surfactants, including TEBS, SDBS, and PS–PEG-COOH, on the photocatalytic HER of **PS**/**PSOS** BHJP under identical conditions (1 mg photocatalyst, 0.2 M AA, 3 wt % H_2_PtCl_6_) (Figure S23b). The results indicate that the presence of additional surfactants inhibits the HER compared to **PS**/**PSOS** BHJP without additional surfactants, likely due to the surfactants interfering with the electron transfer process (Figure 5c). Furthermore, the HER of the **PS**/**PSOS/**surfactant NP decreased even more compared to **PS**/**PSOS** BHJP with additional surfactants, likely due to the stronger interaction of the surfactants with the polymer during the preparation process, which further hindered electron transfer (Figure S25).

Here, the cost of various methods employed to prepare heterojunction particles was calculated, as summarized in Tables S7–S9. The estimated costs for facile precipitation, nanoprecipitation, mini-emulsion using TEBS, and mini-emulsion approach using SDBS are 0.0846, 0.5485, 21.489, and 0.5676 dollars, respectively (Figure 5d). Notably, the facile precipitation method exhibits the lowest cost, attributed to its preparation process not requiring the addition of surfactants, suggesting its significant potential for future large-scale hydrogen production systems. The high cost of the mini-emulsion approach is due to the need for 50 times more polymer weight of surfactant.

Furthermore, we employed femtosecond to nanosecond TAS to investigate the dynamics of charge carriers in heterojunction particles prepared via different methods (Figure S26). To investigate the origin of the PIA signal, kinetic decay analyses were performed on the curves displaying the strongest signals (Figure S27).^40,41^ Using a three-exponential model, we identified two rapid components (τ_1_ and τ_2_) in the picosecond range, attributed to electron transfer to Pt sites, while the long-lived component (τ_3_) was linked to charge carrier recombination.^42,43^**PS**/**PSOS** BHJP exhibited shorter lifetime components (τ_1_ and τ_2_) compared to **PS**/**PSOS/TEBS** NP and **PS**/**PSOS/PS–PEG-COOH** NP suggest an accelerated transfer of photoexcited electrons to Pt sites (Figure 5e). This observation reveals the necessity of surfactants in nanoprecipitation and the mini-emulsion approach for stabilizing the structure. The substantial surfactant, which lacks electron transfer capability, impedes the efficient transfer of electrons between Pt and the polymer, adversely affecting the photocatalytic hydrogen evolution reaction. In addition, we meticulously assessed the population of electron polarons at 740 nm in heterojunction particles prepared via different methods (Figure 5f). **PS**/**PSOS** BHJP exhibited the most prominent polaron signal. In our facile precipitation method, AA was included during the BHJP preparation process, whereas in the nanoprecipitation and mini-emulsion approaches, AA was added after heterojunction particle formation. This resulted in a stronger interaction between **PS**/**PSOS** BHJP and AA in the facile precipitation method, leading to a higher generation of electron polarons (Figure 5g,h).

**Figure 6:**
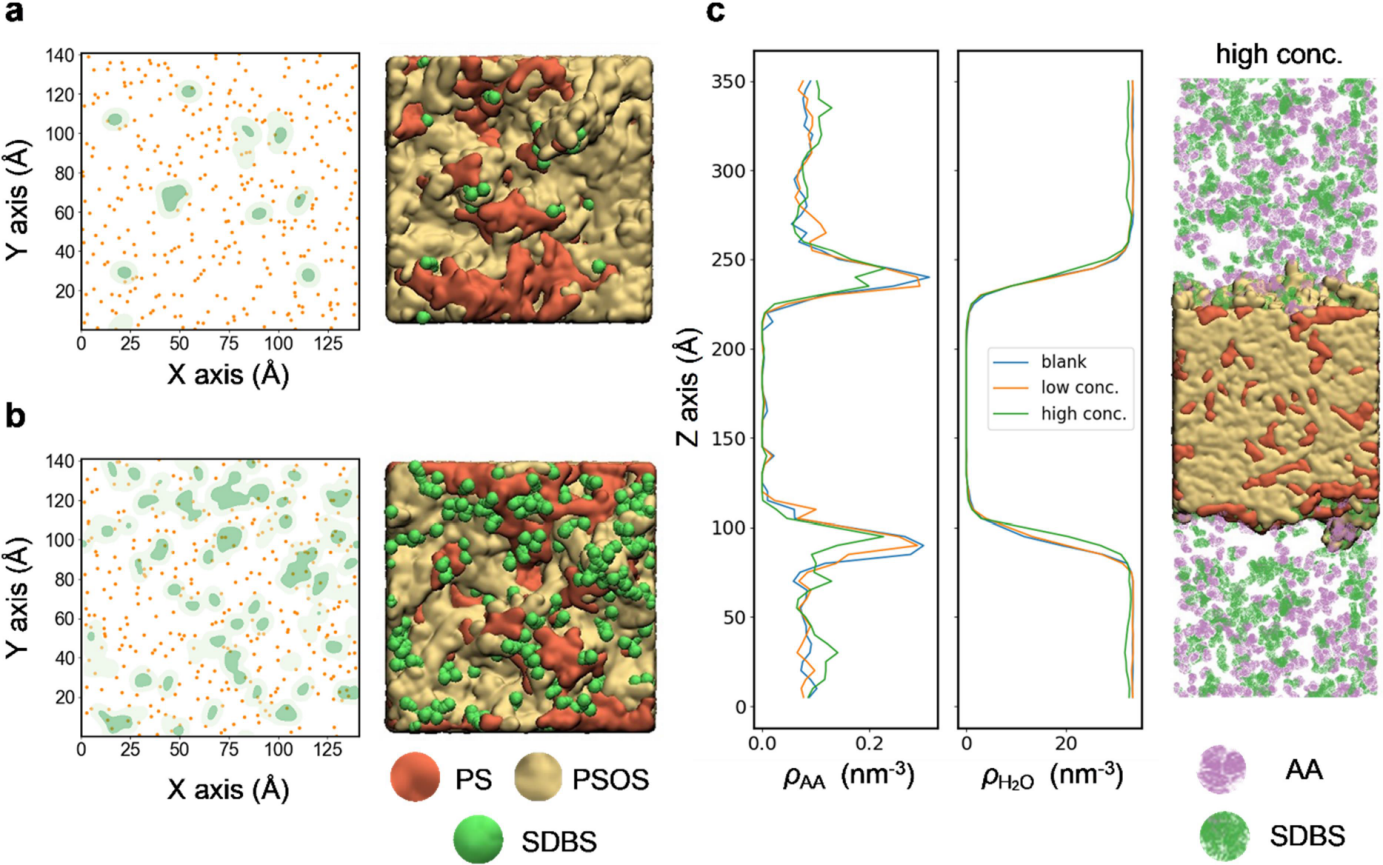
Spatial distribution of chemical species (left) and associated interfacial model generated via MD (right). Sulfone groups (orange dots) and SDBS (green area) near the polymer surface (<2.5 Å) under (a) a low concentration of SDBS and (b) a high concentration of SDBS, (c) Number density profile of AA (ρ_AA_) and water (ρ_H2O_) along the *Z*-axis (normal to the polymer-solution interface) under different SDBS concentrations.

To gain deeper insights into the role of surfactants in photocatalytic hydrogen evolution, we utilized MD simulations to model a polymer blend (1:3)-solution interface, incorporating varying concentrations of SDBS surfactant (Figure S28 and Table S10). Our MD analysis reveals a dual function of the SDBS surfactant. First, as the concentration of SDBS molecules increases, more sulfone groups at the interface are covered by the surfactant (Figure 6a,b). Since sulfone groups are generally considered to be active nucleation sites for cocatalyst,^38^ this obstruction likely reduces the deposition of cocatalyst on the **PS**/**PSOS** BHJP, thereby diminishing the electron transfer efficiency from the polymer to Pt. Second, we observed a decrease in the interfacial concentration of AA with increasing SDBS concentration, which could potentially slow down electron polaron generation (Figure 6c). These findings from our MD simulations align with our TAS observations, supporting our hypothesis regarding the role of surfactants.

Finally, through a combination of density functional theory (DFT) calculations and experimental analysis, the mechanism of photocatalytic hydrogen evolution reaction was elucidated. Upon light absorption, the BHJP facilitate exciton generation, which rapidly dissociates at the **PS**/**PSOS** heterojunction interface, driven by the advantageous type II energy level alignment between the two polymers. Holes are transferred to the donor **PS** phase, while electrons migrate to the acceptor **PSOS** phase. These holes are effectively scavenged by AA, resulting in the formation of long-lived electron polarons on the polymer backbone. The absence of AA significantly diminishes the electron polaron signal, as shown in Figure 4f, highlighting the critical role of AA in sustaining electron polarons. The hydrogen evolution reaction is catalyzed by platinum clusters, with charge transfer from the polymer to the platinum facilitated by sulfone groups in the **PSOS** structure. DFT calculations show that the driving force for hydrogen evolution reaction, defined as the difference between the LUMO energy level of **PSOS** and the absolute potential of H^+^/H_2_, is approximately 0.45 eV at pH 4. However, the Gibbs free energy change for proton adsorption (|Δ*G*_h_*|) on **PSOS** is 1.52 eV, which exceeds the available driving force (Figure S29). By contrast, platinum exhibits a |Δ*G*_h_*| of 0.09 eV, well within the driving force, affirming its role as the active catalytic site. These findings indicate that the BHJP primarily serves as a photosensitizer, facilitating exciton dissociation and charge transport, while hydrogen evolution reaction occurs predominantly on the platinum surface (Figure S30).

## 3 Conclusions

In summary, we have introduced a facile precipitation method for fabricating BHJP utilizing the donor polymers **PS** and the acceptor polymer **PSOS**. Unlike previously reported techniques such as mini-emulsion and nanoprecipitation, this novel methodology offers versatility and several advantages, including simplicity in fabrication, low cost, absence of post-treatment requirements, and bare-to-use surfactants. The exceptional structural similarity between the donor and acceptor polymers enhances their miscibility, effectively mitigating phase separation and promoting intermixed domains with substantial D–A interfaces within the blends. Furthermore, SAXS and USAXS analyses reveal that the BHJP form cross-linked networks in aqueous solution, characterized by distinct mass fractal dimensions and aggregation of small polymer spherical particles. The **PS**: **PSOS** (1:3) BHJP achieved a maximum HER of 251.2 mmol h^–1^ g^–1^ and an AQY of 26.2% at 500 nm. Compared to other methods, the facile precipitation technique delivers an HER improvement of 218–1821%. Transient absorption spectroscopic studies revealed that the absence of surfactants in this method accelerates electron transfer to the cocatalyst and promotes electron polaron formation. MD simulations further confirmed that surfactants block sulfone groups, crucial nucleation sites for cocatalyst, reducing cocatalyst deposition and impeding electron transfer. Surfactants also decrease the interfacial AA concentration, potentially slowing electron polaron generation. These factors collectively contribute to the superior HER observed in this innovative methodology, propelling the field of solar hydrogen generation forward and addressing limitations inherent in existing techniques. This advancement provides a sturdy groundwork for the development of sustainable and highly efficient energy conversion technologies.

## Supporting Information Available

Experimental section, including materials, general methods, contact angle measurements and interfacial parameter calculation, mini-emulsion and nanoprecipitation and facile precipitation method for heterojunction material fabrication and HER, AQY measurement, MD simulation, hydrogen adsorption calculations, SAXS measurement and data fitting, and TAS measurement (PDF)
Photocatalytic hydrogen evolution process of PS:PSOS(1:3)BHJP (MP4)
Photocatalytic hydrogen evolution process of a mixture of PS and PSOS pristine particles (MP4)
