# Supplementary material for: Optimally Miscible Polymer Bulk-Heterojunction-Particles for Nonsurfactant Photocatalytic Hydrogen Evolution
Source: J Am Chem Soc. 2024 Dec 20;147(3):2537–48. doi: 10.1021/jacs.4c13856 (PMC11760146; doi:10.1021/jacs.4c13856)
Supplement: Supplementary file 1 — ja4c13856_si_001.pdf [file ja4c13856_si_001.pdf]

Supporting Information

Optimally Miscible Polymer Bulk-Heterojunction-Particles for NonSurfactant Photocatalytic Hydrogen Evolution

Wei-Cheng Lin[a], Yu-En Sun[a], Ying-Rang Zhuang[a], Tse-Fu Huang[a], Kuei-Jhong Lin[a], Mohamed M. Elsenety[a,b], Jui-Chen Yen[c], Hung-Kai Hsu[c], Bo-Han Chen[c], Chen-Yu Chang [a,d], Je-Wei Chang[d], Hsin-Ni Huang[a], Bing-Heng Li[a], Siriporn Jungsuttiwong[e], Toton Haldar[f,g], Shin-Huei Wang[f], Wan-Chi Lin[f], Tien-Lin Wu[h], Chin-Wen Chen[g], Chi-Hua Yu[f], An-Chung Su[a], Kun-Han Lin[a,], U-Ser Jeng[a,d,j,]*, Shang-Da Yang[c,i,j,]*, and Ho-Hsiu Chou[a,i,j,]**

[a.]Department of Chemical Engineering, National Tsing Hua University, Hsinchu 300044, Taiwan

[b.]Department of Chemistry, Faculty of Science, Al-Azhar University, Nasr City, Cairo, 11884, Egypt

[c.]Institute of Photonics Technologies & Department of Electrical Engineering, National Tsing Hua University, Hsinchu 300044, Taiwan

[d.]National Synchrotron Radiation Research Center, Hsinchu 30076, Taiwan

[e.]Department of Chemistry and Center of Excellence for Innovation in Chemistry, Faculty of Science, Ubon Ratchathani University, Ubon Ratchathani 34190, Thailand

[f.]Department of Engineering Science, National Cheng Kung University, Tainan 701401, Taiwan

[g.]Department of Molecular Science and Engineering, National Taipei University of Technology, Taipei 106344, Taiwan

[h.]Department of Chemistry, National Tsing Hua University, Hsinchu, 300044 Taiwan

[i.]Center for Photonics Research, National Tsing Hua University, Hsinchu 300044, Taiwan

[j.]College of Semiconductor Research, National Tsing Hua University, Hsinchu 300044, Taiwan

* Corresponding author. E-mail: kunhan.lin@mx.nthu.edu.tw; usjeng@nsrrc.org.tw ; shangda@ee.nthu.edu.tw ; hhchou@mx.nthu.edu.tw

Keywords: Facile precipitation method; surfactant-free bulk-heterojunction particles; high molecular compatibility; polaron generation; photocatalytic hydrogen evolution

Experimental Section

Materials

All reagents were sourced from commercial suppliers and used without additional purification. Ascorbic acid was obtained from Acros Organics. **PS** and **PSOS** were synthesized using the Pd-catalyzed Suzuki–Miyaura coupling method. The molecular weight of **PS** and **PSOS** were determined by gel permeation chromatography. The number-average molecular weights (M_n) of the PS and PSOS were 9111 and 6668 g mol^{-1}, weight-average molecular weights (M_w) were 19053 and 14260 g mol^{-1}. The solid-state UV–vis absorption spectra of two polymers were measured, and optical bandgap values were determined using the Tauc plot method. The optical bandgaps of **PS** and **PSOS** were calculated as 2.07 eV and 2.10 eV, respectively.Ultraviolet photoelectron spectroscopy was employed to determine the HOMO energy levels, while the LUMO levels were derived by subtracting the bandgap values from the HOMO levels. The HOMO/LUMO energy levels for **PS** and **PSOS** are -5.34/-3.27 eV and -5.85/-3.75 eV, respectively. It note that all data presented here, including molecular weights, optical bandgap, and energy levels, were derived from our laboratory's previous studies and are consistent with the materials and methodologies used in prior work.[1] **PM6** and **Y6** were procured from SunaTech Inc. TEBS was acquired from Solaris Chem. PS-PEG-COOH was purchased from Polymer Source.

General methods

Fourier-transform infrared spectra were obtained with a Thermo Scientific iS50 spectrometer. UV–vis absorption spectra of the polymers were measured using a Hitachi U-3300 spectrophotometer. X-ray photoelectron spectra were acquired using a ULVAC-PHI PHI Quantera II system. Water used for the HER experiments was purified with an ELGA LabWater system. The hydrodynamic diameter of the heterojunction particles was determined using a Zetasizer Nano ZS90 (Malvern Instruments Nordic AB). Transmission electron microscopy images were acquired with a JEM-2100(HT) 200 kV electron microscope (LaB6) equipped with 3D tomography capabilities.

Synthesis of PS and PSOS

A round-bottomed flask was charged with co-monomers, Na_2CO_3, tetra-n-butylammonium bromide

(TBAB), and $Pd(PPh_3)_4$, along with toluene and water as solvents. The reaction mixture was degassed by bubbling nitrogen for 30 minutes and refluxed at 120 °C for 48 hours. After cooling to room temperature, the reaction mixture was poured into methanol to precipitate the polymer. The precipitated polymer was collected by membrane filtration and purified via Soxhlet extraction using methanol, hexane, and water. The polymer was then dried under vacuum.

Contact angle measurements and interfacial parameter calculation

The contact angles of materials were measured using a contact angle analyzer (First Ten Angstroms, FTA1000B). The surface energy of thin films was determined through the Wu model, utilizing contact angles from water and ethylene glycol on the films. The compatibility between the two components was assessed by the Flory–Huggins interaction parameter equation $\chi_{A\text{-}B} = K[(\gamma_A{}^{1/2})\text{-}(\gamma_B{}^{1/2})]^2$.[2]

Mini-emulsion method for BHJP fabrication and photocatalytic hydrogen evolution experiments

Individual stock solutions (1 mg mL^{-1}) of **PS** and **PSOS** were prepared in chloroform. Precursor solutions were then created by blending the stock solutions in the desired particle composition ratio and subjected to sonication for 10 minutes to ensure homogeneous mixing of the two polymers. Subsequently, 1 mL of the precursor solution was added to 50 mg of surfactant (TEBS) in 10 ml of water and sonicated for 30 minutes using an ultrasonic processor to generate a mini-emulsion. The resulting mini-emulsion was heated at 85 °C under air to eliminate the chloroform for one hour, resulting in a surfactant-stabilized BHJP dispersion in water. Subsequently, AA and H_2PtCl_6 were introduced, and the pH was adjusted to 4.00 using 10 M KOH. The mixture underwent further degassing with argon for 10 minutes, was maintained under negative pressure, and exposed to illumination using a 350 W Xe lamp (1000 W m^{-2}, λ= 380-780 nm) at room temperature. The quantity of hydrogen produced was determined using gas chromatography with a thermal conductivity detector, with standard hydrogen gas as a reference.

Nanoprecipitation method for BHJP fabrication photocatalytic hydrogen evolution experiments

Individual stock solutions (1 mg mL^{-1}) of **PS** and **PSOS** were prepared in tetrahydrofuran (THF). Precursor solutions were then created by blending the stock solutions in the desired particle composition ratio and subjected to sonication for 10 minutes to ensure thorough mixing of the two polymers. Subsequently, 1 ml

of the precursor solution and PS-PEG-COOH (1 mg mL^{-1} in THF, 1 mL) were mixed with THF (5 mL). This solution was rapidly injected into water (10 mL) under vigorous sonication for 5 minutes. The resulting mixture was kept under negative pressure to remove THF for one hour, yielding a surfactant-stabilized BHJP dispersion in water. Subsequently, AA and H_2PtCl_6 were introduced, and the pH was adjusted to 4.00 using 10 M KOH. The mixture underwent further degassing with argon for 10 minutes, was maintained under negative pressure, and exposed to illumination using a 350 W Xe lamp (1000 W m^{-2}, λ= 380-780 nm) at room temperature. The quantity of hydrogen produced was determined using gas chromatography with a thermal conductivity detector, with standard hydrogen gas as a reference.

BHJP fabrication and photocatalytic hydrogen evolution experiment

Initially, **PS** and **PSOS** powders with varying **PS**/**PSOS** ratios (0.5, 1, 2 mg) and 1 mL of NMP were placed in a reaction glass, followed by sonication for 10 minutes to ensure the complete dissolution of polymers. Subsequently, 9 mL of DI water with 0.2 or 1 M AA was added to the glass, rapidly forming BHJP. 3wt% H_2PtCl_6 was introduced, and the pH was adjusted to 4.00 using 10 M KOH. The mixture underwent further degassing with argon for 10 minutes, was maintained under negative pressure, and exposed to illumination using a 350 W Xe lamp (1000 W m^{-2}, λ= 380-780 nm) at room temperature. The quantity of hydrogen produced was determined using gas chromatography with a thermal conductivity detector, with standard hydrogen gas as a reference.

AQY measurement

AQY measurements were conducted similarly to hydrogen evolution assessments, incorporating band-pass filters (420, 460, 500, 550, 600 nm) on the light source. The AQY for hydrogen evolution is defined as the ratio of the number of reacted electrons to the number of incident photons, assuming a two-electron process for the entire reaction. The irradiation area and time were 6 cm^2 and 3600 s, respectively. The AQY was calculated using the following equations:

AQY = (2 × amount of reacted electrons amount of incident photons) × 100%

$$= (2 \times \mathrm{m} \times \hbar \times N_A) / (\mathrm{A} \times P \times t \times \lambda / \mathrm{c}) \times 100\%,$$

where m is the amount of hydrogen produced (mol), $\hbar$ is the Planck constant (J s), NA is the Avogadro constant (mol^{-1}), A is the irradiation area (m^2), P is the intensity of the monochromatic light ($W\ m^{-2}$), t is the time (s), λ is the wavelength of the monochromatic light (m), and c is the speed of light ($m\ s^{-1}$).

Computational Section

General setting in MD simulation

All the MD simulations were performed using GROMACS of version 2019.6.[3] Periodic boundary conditions (PBC) were applied in all three dimensions (xyz), and long-range electrostatic interactions were calculated using the particle mesh Ewald (PME) method with a cutoff radius of 1.3 nm for non-bonded interactions. Temperature was controlled using velocity rescaling with a stochastic term and a temperature coupling constant (τ_T = 0.5 ps). The pressure was adjusted using the Berendsen barostat through semi-isotropic coupling with a compressibility of $\chi = 4.5 \times 10^{-5}$ bar^{-1} and a pressure coupling constant (τ_P = 0.5 ps).

Forcefield parameterization

We used Q-Force to perform the Hessian fitting protocol to parameterize the bonded parameters, including proper and improper dihedrals, as well as other bonded interactions. Electron densities were calculated using Gaussian16 at the ωB97X-D32/6–311 G(d,p) level of theory. Non-bonded parameters, such as atomic partial charges and Lennard-Jones parameters, were derived following the method proposed by Cole et al.[4] Briefly, overlapping atomic electron densities were obtained using the density-derived electrostatic and chemical (DDEC6) electron density partitioning scheme,[5] and atomic partial charges were then determined by integrating these densities over the entire space. The parameters A and B for the Lennard-Jones potential were derived using the Tkatchenko−Scheffler (TS) scheme.[6] DDEC6 computations were carried out using Chargemol version 09_26_2017. After setting the non-bonded parameters, flexible dihedrals were parameterized through constrained optimization scans performed at the ωB97X-D/6-311G(d,p) level using Gaussian16. For further details on the parameterization of dihedral potentials. [7]

Polymer solid bulk generation (PS / PSOS / PS+PSOS)

We constructed three distinct polymer bulk structures, as shown in **Fig. 3**. These structures differ in the composition of polymer chains, with the following proportions: 1000 polymer chains of **PS**, 1000 polymer chains of **PSOS**, and a polymer blend structure comprising 500 polymer chains of **PS** and 500 polymer chains of **PSOS**. Each polymer chain consists of 4 repeat units. Figure SA shows the flowchart of polymer

bulk construction. Initially, we placed 1000 polymer chains into the simulation box at an approximate density of 15 kg/m³ using Packmol.[8] We then performed energy minimization, followed by a compression process at a pressure of 50 bar and a temperature of 300 K until the system's density reached 250 kg/m³. The system was then heated from 300 K to 800 K at a rate of 1 K/ps, followed by a 10 ns equilibration at 800 K. Finally, the system was quenched back to 300 K at a cooling rate of 1 K/ps, and an 80 ns equilibration at 300 K was conducted to ensure that the potential energy of the system reached equilibrium.

Solubility parameters and Flory-Huggins theory

To calculate the solubility and Flory-Huggins parameters from the MD simulation results, we followed the protocol proposed by Zhonglin Luo et al.[9] The solubility parameter (δ) is given by:

$$\delta = \sqrt{\frac{E_{coh}}{V}} = \sqrt{\frac{E_{vac} - E_{bulk}}{V}} = \sqrt{CED}$$

where CED represents the cohesive energy density. We averaged the potential energy from the last 100 ps of the cooling process during polymer bulk construction to obtain bulk potential energy (E_{bulk}). Vacuum potential energy (E_{vac}) was calculated using a single-chain MD simulation run under NVT conditions at 300 K for 500 ps. These potential energy values and the results are shown in **Table S2**. The experimental solubility parameter (δ_{exp}) was calculated according to the method described in the work by Kouijzer, Sandra, et al.,[10] where the proportional constant K was chosen to be 118 (as shown in **Table S2**).

The Flory-Huggins parameter can be calculated using two different approaches: either with or without considering the mixing potential energy. The parameter χ without considering the mixing potential energy is given by:

$$\chi = \frac{V_{mono}}{RT}(\delta_A - \delta_B)^2$$

where V_{mono} was calculated by dividing the total volume of the mixture by the number of repeat units in the system. This yields a value of $\chi = 0.366$. The parameter χ_{mix}, which considers the mixing potential energy, is given by:

$$\chi = \left(\frac{\Delta E_{mix}}{RT}\right) V_{mono}$$

ΔE_{mix} is the energy change of mixing per unit volume, which is given by:

$$\Delta E_{mix} = \phi_A \left(\frac{E_{coh}}{V}\right)_A + \phi_B \left(\frac{E_{coh}}{V}\right)_B - \left(\frac{E_{coh}}{V}\right)_{mix}$$

We analyzed two scenarios with polymer **PS** volume fractions (ϕ_{PS}) of 0.5 (1:1) and 0.25 (1:3). The calculated χ_{mix} values were similar, at 0.399 for the 1:1 case and 0.415 for the 1:3 case.

Hydrogen Adsorption Calculations

To determine whether PSOS acts as an active HER catalyst, we evaluated the hydrogen adsorption free energy (ΔG_{H^*}) for several potential adsorption sites. A monomer model of PSOS, excluding long alkyl chains, was used to reduce computational costs. The ΔG_{H^*} values for various adsorption sites and their configurations are presented in **Supplementary Fig. 14**. The ΔG_{H^*} was calculated using the following equation:

$$\Delta G_{\mathrm{H}^*} = G_{\mathrm{H}^*,(aq)} - G_{*,(aq)} - \frac{1}{2} G_{\mathrm{H}_2,(g)}$$

where $G_{H^*,(aq)}$ is the free energy of the polymer with adsorbed hydrogen in aqueous solution, $G_{*,(aq)}$ is the free energy of the polymer without hydrogen in aqueous solution, and $G_{H_2,(g)}$ is the free energy of hydrogen gas. The calculations were conducted using Gaussian16 at the M08-HX/6-311G(d,p) level of theory. The SMD implicit solvation model was employed with water as the solvent to simulate the aqueous environment during HER.

In addition, we computed the ΔG_{H^*} for Pt, which is used as a co-catalyst in this study. A 3x3 Pt (111) 5-layer slab model was constructed for this computation, and its ΔG_{H^*} is also shown in **Supplementary Fig. 14.** The calculations were performed using DFT with the Perdew-Burke-Ernzerhof (PBE) exchange-correlation functional, as implemented in the Vienna Ab-initio Simulation Package (VASP). Valence electron wave functions were represented using a plane-wave basis set with a cutoff energy of 400 eV, and core-electron interactions were treated with the projector-augmented wave (PAW) method. The Brillouin zone was sampled using a Γ-centered 4 × 4 × 1 k-point grid. Structural optimizations were carried out until the residual forces on all atoms were reduced to below 0.02 eV $Å^{-1}$.

Polymer-solution mixture simulation

To study the interaction of the surfactant and photocatalysts, we generated a polymer blend using a total of 500 polymer chains, with a **PS**:**PSOS** (1:3), which is the optimal performance ratio mentioned in the literature. We placed the polymer chains into the simulation box using Packmol and then generated the bulk structure following similar procedures as mentioned above. The only difference in the procedure is that the cooling equilibration time was reduced to 29.5 ns due to the smaller system size.

Next, we prepared solutions under three different SDBS concentration conditions, with the detailed concentrations of each solvent shown in **Table S9**. The low concentration condition was designed to mimic experimental concentration conditions, where the simulation box contained a total of 134,000 water molecules, along with 510 AA molecules and 52 SDBS molecules. We set the initial solution density to 1000 kg/m^3. A flowchart of polymer-solution MD simulation is presented in **Fig. S14**. Initially, we used Packmol to place solution molecules above the top surface of the 500-chain polymer bulk at an approximate distance of 20 Å. We then performed energy minimization, followed by a 5 ns NVT simulation at 300 K. After that, we conducted an NPT simulation at 50 bar and 300 K for 1 ns. Finally, we ran an additional 21 ns NPT equilibration at 300 K and 1 bar.

Small-Angle X-Ray Scattering

SAXS and USAXS data for the solution samples were measured using the TPS13A BioSWAXS beamline at the National Synchrotron Radiation Research Center.[11] Samples were loaded into thin-wall quartz capillaries for X-ray scattering measurements. The X-ray detectors used at the experimental station were Eiger X 9M and X 1M, with a pixel size of 75*75 μm. The active areas are 3109*3268 pixels and 1029*1064 pixels, respectively. Eiger X 9M primarily received data from small-angle scattering. TPS 13A could greatly reduce air molecule scattering background noise and cover data in a wide q-range with the two-detector system in a vacuum. The incident X-ray beam energy was 15 keV, corresponding to a wavelength (λ) of 0.827 Å. The sample-to-detector (SD) distance is 2.5 m for the Eiger X 9M detector and 0.25 m for the Eiger X 1M detector, together covering a q-range from 0.006 to 2.5 $Å^{-1}$. Additional SD distance of Eiger X 9M enabled a broader q-range to be observed. This study adjusted Eiger X 9M for SD =10.0 m and 2.5 m, covering a SAXS q-range of 0.0014 - 0.6 $Å^{-1}$. The SAXS data were collected at a frame rate of 2 frame/s for 5-10 frames. The sample and buffer transmission coefficients were measured for buffer scattering subtraction.

Model for SAXS data fitting

SAXS intensity profiles $I(q)$ for the **PS**/**PSOS** bulk heterojunction particles (BHJP) were fitted using a fractal model comprising polymer particles. The structural characteristics described by this model involve the aggregation of the primary spherical particles into fractal-like aggregation structures, as defined by the scattering:

$$I(q) = \Psi V_p(\rho_B - \rho_S)^2 P(q)S(q) \quad (1)$$

where Ψ represents the volume fraction occupied by the primary particles, and V_P denotes the volume of the scattering particles defined by spheres in this context; ρ_B and ρ_S respectively represent the electron scattering length densities (SLD) of the spherical particles and the solvent. Their SLD difference of $(\rho_B - \rho_S)$, scattering contrast, dominates the SAXS intensity. In Equation (1), $P(q)$ represents the normalized form factor and $S(q)$ the structure factor,[12] with the scattering vector $q = 4\pi\lambda^{-1}\sin\theta$ defined by the X-ray wavelength and the scattering angle 2θ. The scattering form factor of spheres of radius r_d is given as follows:

$$P(q) = F(qr_d)^2 \qquad (2)$$

$$F(x{=}qr_d) = \frac{3[\sin(x) - x\cos(x)]}{x^3} \qquad (3)$$

The structure factor of a mass fractal system comprising spherical particles is described by

$$S_f(q) = 1 + \frac{D_f \Gamma(D_f - 1)}{\left[1+\frac{1}{(q\xi)^2}\right]^{(D_f-1)/2}} \frac{\sin[(D_f - 1)tan^{-1}(q\xi)]}{(qR_e)^{D_f}} \qquad (4)$$

D_f represents the fractal dimension, indicating the self-similarity of the system or the degree of clustering in the structure.[12] R_e denotes the effective radius (= r_d in this study) of the building blocks of the fractal aggregates, while ξ is the correlation length, indicating a characteristic size of irregular aggregates in the system. The total size length can be roughly estimated from the two times of R_g deduced from the correlation length, with $R_g = [D_f(D_f{+}1)/2]^{1/2}\xi$. Effective radius and correlation length represent the scales of minimum and the maximum lengths of the system, respectively. Lengths within this range are manifested in the scattering data in a power-law form. This mass fractal model comprising primary spherical particles is found to adequately descerib percolation systems in the solution. [12]

Transient Absorption Spectroscopy Setup

Transient absorption spectroscopy was conducted using a commercial Yb:KGW laser system (Pharos, Light Conversion) operating at a central wavelength of 1030 nm. The system provided 2.5 W of average power, a repetition rate of 3.125 kHz, pulse energy of 800 μJ, and a pulse duration of 190 fs. A low-dispersion 50/50 beam splitter generated twin pulses, which were compressed using a custom nonlinear compressor based on the multiple plate compression (MPC) technique. A high-pass filter with a cut-off at 980 nm removed low-frequency noise. Pulse compression was optimized using eight chirped mirrors (Ultrafast Innovation) to compensate for dispersion introduced by the optical elements. The compressed pulses, characterized by polarization-gating frequency-resolved optical gating (PG-FROG), achieved a full width at half maximum (FWHM) of 3.2 fs at the sample position. Pump pulse modulation was managed by a laser-triggered mechanical chopper operating at half the laser's repetition rate (1.5625 kHz). A combination of tunable high-pass and low-pass filters adjusted the pump bandwidth and central wavelength. Power was precisely controlled using a broadband half-wave plate and wire-grid polarizer to prevent nonlinear effects. A linear translation stage (DL325, Newport) provided a delay range of up to 2.2 ns between the pump and probe pulses. The beams were focused on the sample at a 5-degree non-collinear angle, with the pump spot size (~67.7 μm) slightly larger than the probe spot (~27.3 μm) to ensure uniform excitation. After interacting with the sample, the transmitted probe beam was separated and analyzed with a custom spectrometer equipped with a high-speed linear array camera (Glaz Linescan-I-Gen2, Synertronic with S12198-512Q CMOS, Hamamatsu), capturing each probe pulse. The $\Delta T/T$ signal was extracted by modulating the pump at half the repetition rate, with spectral differences between consecutive probe pulses providing the contrast needed for measurement.

Deconvoluted PIA signals of exciton and polaron

The deconvolution of PIA signals from exciton and polaron components proceeded as follows: Initially, the TAS captured at 1 ps post-excitation was presumed to solely manifest spectral signatures attributable to exciton absorption (positive, spanning 600-1000 nm). This temporal point sufficiently mitigated instrument response artifacts while precluding substantial contributions from charge formation. Subsequently, for all

spectra beyond 1 ps (each treated as "the spectrum of interest"), the 1 ps spectrum was proportionally adjusted to align its absorption amplitude at 900 nm (the peak amplitude of the PIA signal) with that of the spectrum of interest. Consequently, this scaled 1 ps spectrum encapsulated both the morphology and the absorption amplitude of all non-polaronic states. Through the subtraction of each scaled 1 ps spectrum from its corresponding spectrum of interest, the absorption contribution stemming from generated polarons was isolated for each temporal instance, notably peaking in the 740 nm range. By combining all spectra together, the kinetics of the electron polaron were extracted. [13]

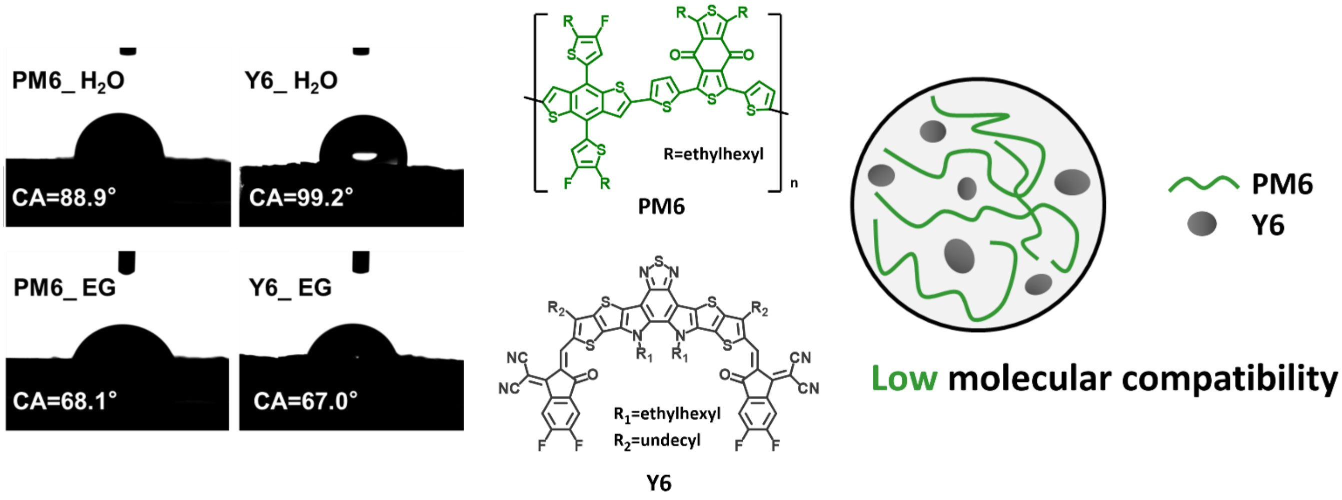

Supplementary Fig. 1. Contact angles with water and ethylene glycol, chemical structures, and compatibility of **PM6** and **Y6**

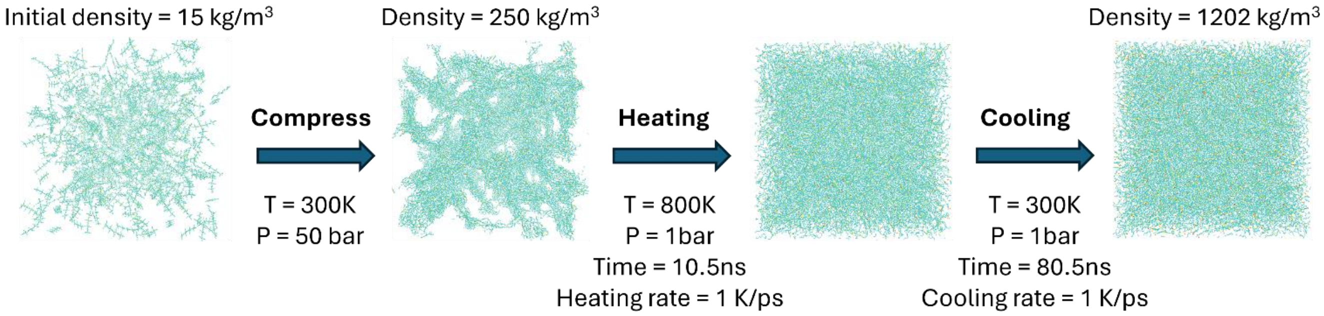

Supplementary Fig. 2. Flowchart of constructing structural models of amorphous polymers

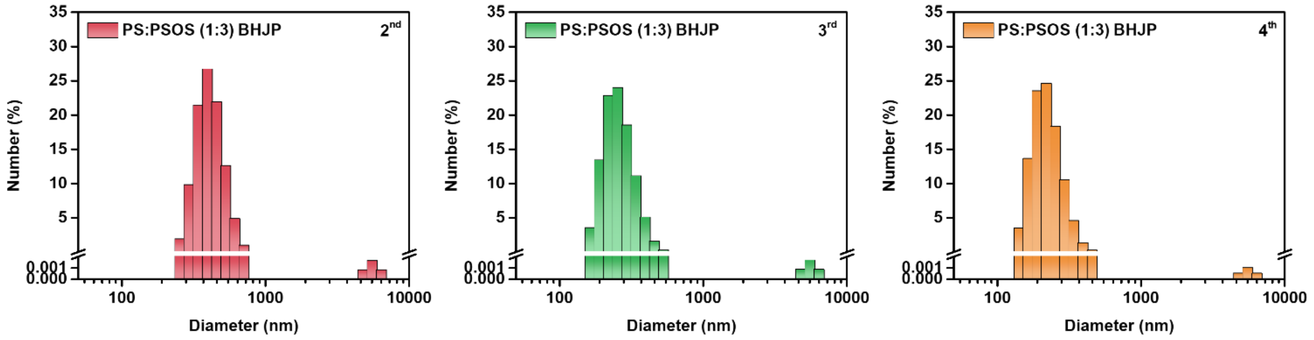

Supplementary Fig. 3. Reproducibility analysis of DLS data for **PS**:**PSOS** (1:3) BHJP

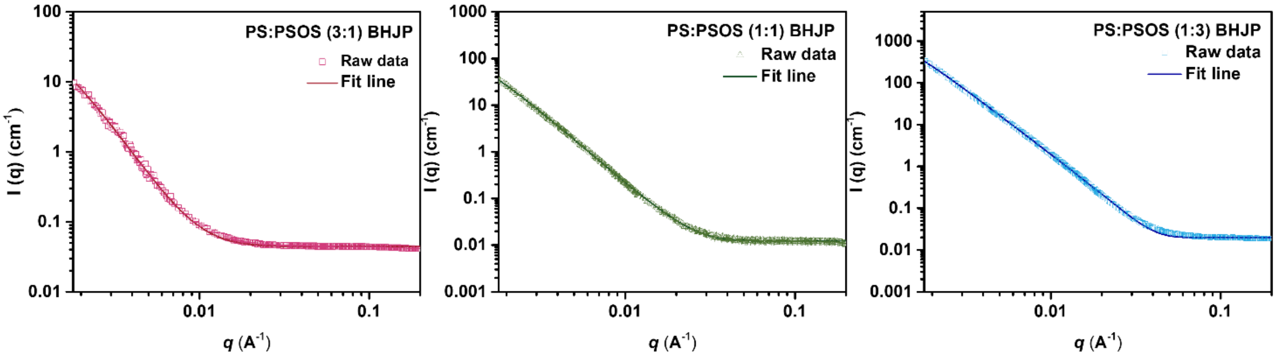

Supplementary Fig. 4. SAXS measurements for the solutions of **PS/PSOS** prepared in different mixing ratios as indicated (of a same sample wt% concentration). The data are fitted (solid curves) using the same fractal model comprising the primary particles of radius r_d, fractal dimension D_f, and cut-off length x. The fitted values are summarized in **Table S4**.

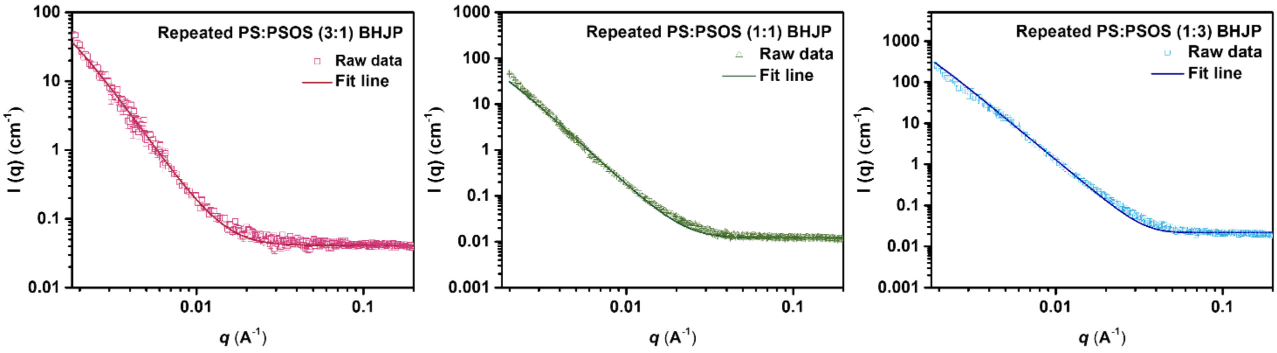

Supplementary Fig. 5. Repeated SAXS measurements for the solutions of **PS/PSOS** prepared in different mixing ratios as indicated (of a same sample wt% concentration). The data are fitted (solid curves) using the same fractal model comprising the primary particles of radius r_d, fractal dimension D_f, and cut-off length x. The fitted values are summarized in **Table S5**.

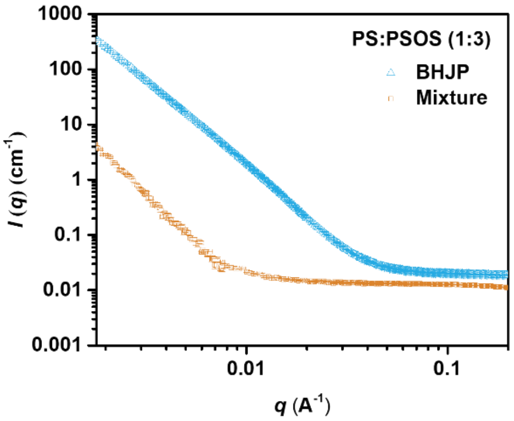

Supplementary Fig. 6. SAXS data for **PS/PSOS** BHJP and pristine particle mixture formed from the **PS/PSOS** ratio of 1:3.

Discussion on Polymer chain arrangement.

To reveal more insight into the formation mechanism of BHJP, we have further measured the SAXS data for the individual components of **PS** and **PSOS** in similar sample concentrations as that used in the BHJP sample solution of **PS**:**PSOS** (1:3). As shown below, the SAXS intensity $I(q)$ profiles of the polymer solutions, with the scattering vector q, can be described adequately using the scattering model for polymer gel or chain networks,[14-15]

$$I(q) = I_F(0)q^{-n} + I(0)/\{1+[(D+1)/3]\xi^2q^2\}^{D/2} \quad (5)$$

Here, $I_F(0)$ and $I(0)$ are the zero-angle scattering intensities of the first and second terms, respectively. The first term describes the power law scattering from large clusters with the exponent n in the low q regime; the second term of a generalized Ornstein-Zernike equation, describes the scattering from polymer chains packed with a fractal dimension D in a length scale characterized by a correlation length ξ (characterizing the fractal domain size).[1,2] As shown in **Fig. S7**, both polymer solutions exhibit common power-law scattering with n = 3.5 in the low q range (0.0015 – 0.0035 Å^{-1}), revealing scattering from the surfaces of solid-like large aggregates. In the higher q region (> 0.01 Å^{-1}), data are fitted with D = 2.0 and ξ = 6.3 nm for the PS-OS polymer solutions, implying smaller network-clusters of Gaussian-chain-like morphology in a theta solvent.[3] Whereas a higher value of D = 2.4 and a similar size of ξ = 5.8 nm fitted for the **PS** solution, suggest that similar networks cluster sizes with denser chain packing of the more hydrophobic **PS** chains. We note that D = 1.67 for linear polymer chains in a good solvent.[4] For comparison, the SAXS intensity

profile of the BHJP solution of **PS**:**PSOS** (1:3) can be described well by of a fractal model covering a wide q-range (0.0015 – 0.04 Å^{-1}), with a larger fractal dimension of $D_f = 2.75$ and a large correlation length of ξ = 106 nm for the fractal networks comprising primary small polymer clusters of a radius of 6.3 nm. The larger D_f and ξ values of **PS**/**PSOS** BHJP, compared to that of either **PS** or **PSOS**, revealing stronger interactions of the two components than the self-affinity for the denser and larger polymer networks. The result is consistent with the MD simulation that predicts complexation formation of **PS**/**PSOS** BHJP.

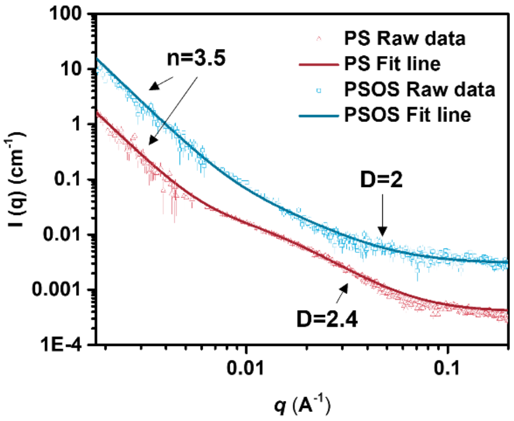

Supplementary Fig. 7 USAXS-SAXS data for the **PS** and **PSOS** solutions of the same sample concentrations as that used in the formation of **PS**/**PSOS** BHJP. The data are fitted using the model for polymer chains as detailed above

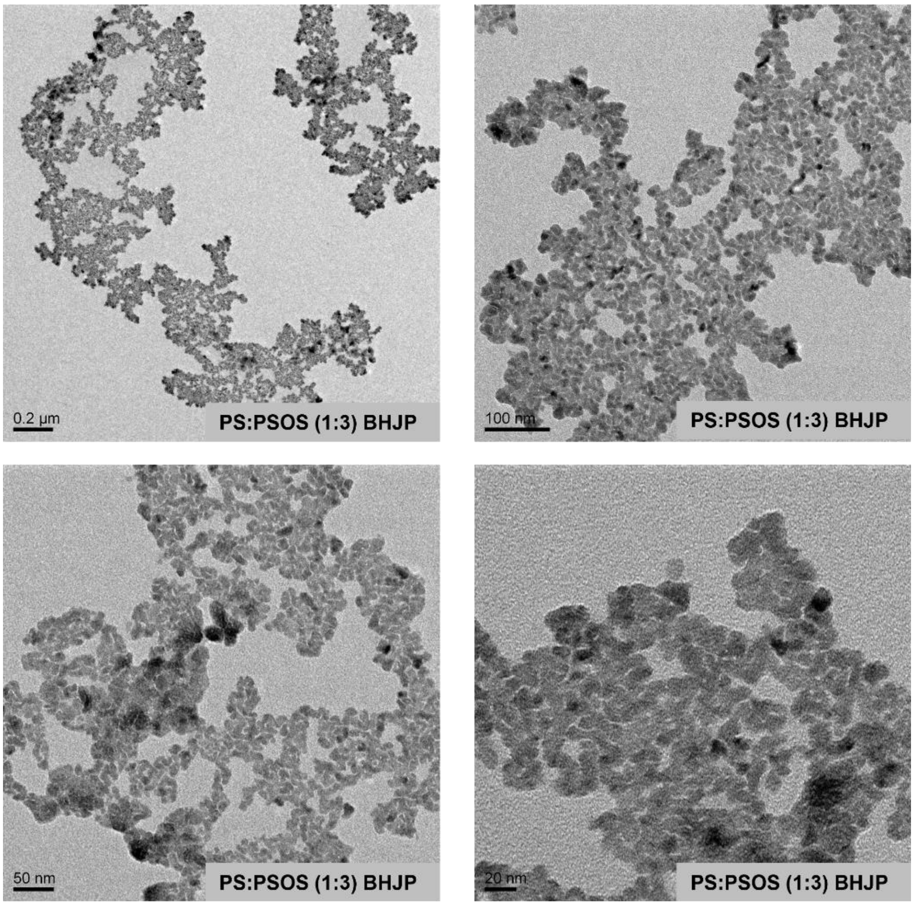

Supplementary Fig. 8. TEM images of the **PS**:**PSOS** (1:3) BHJP at different magnifications

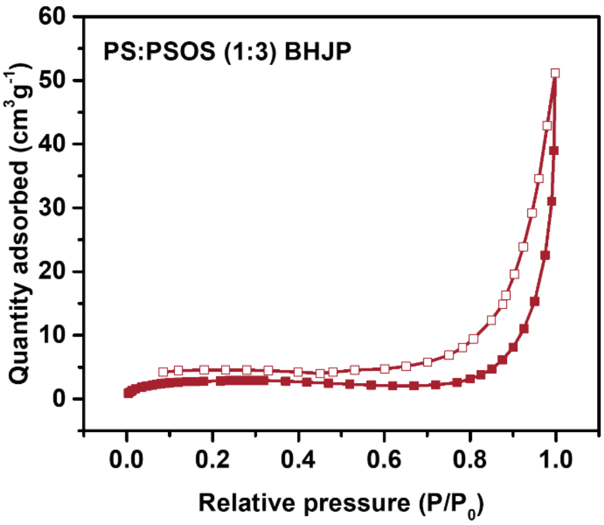

Supplementary Fig. 9. Nitrogen adsorption/desorption isotherms for the **PS**:**PSOS** (1:3) BHJP

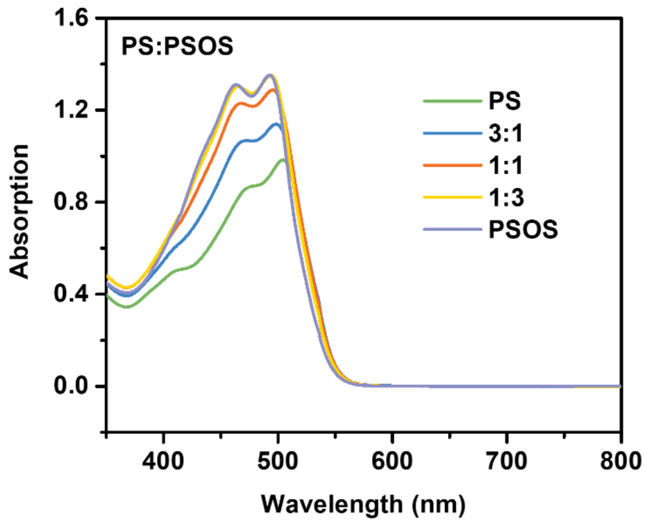

Supplementary Fig. 10 Absorption spectra of **PS**/**PSOS** blends in THF solvent at various ratios. UV-Vis absorption spectra were measured using polymer solutions prepared at a concentration of 0.1 mg mL^{-1} in THF.

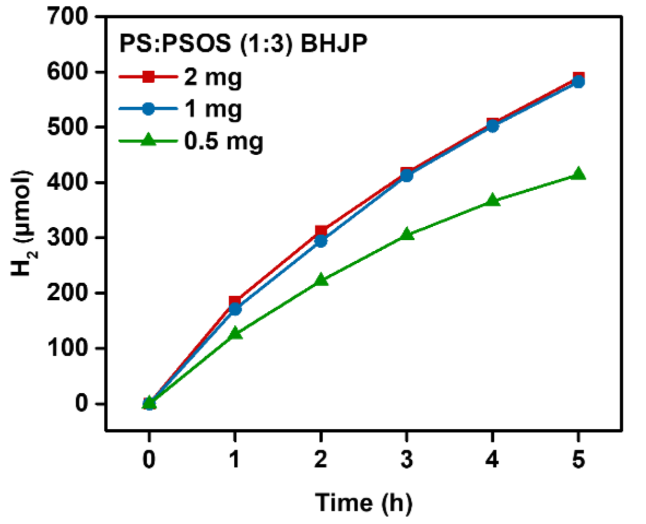

Supplementary Fig. 11 HER of **PS**:**PSOS** (1:3) BHJP at different photocatalyst dosages (2/1/0.5 mg). Conditions: 10 mL water/NMP (9:1 v/v), 1 M AA, and 3 wt% H_2PtCl_6

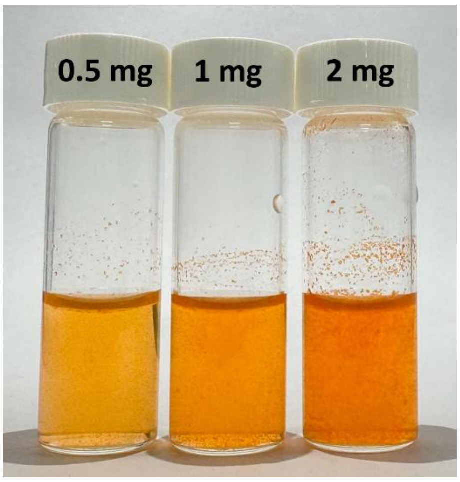

Supplementary Fig. 12 Images of **PS:PSOS** (1:3) BHJP at different polymer dosage

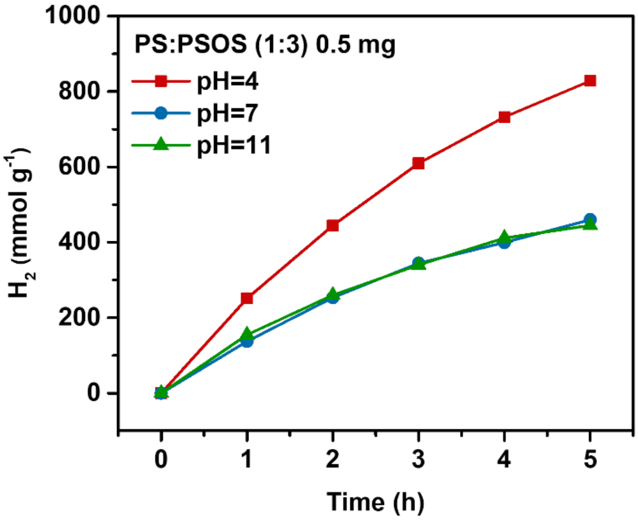

Supplementary Fig. 13 HER of **PS:PSOS** (1:3) BHJP at varying pH value. Conditions: 0.5 mg photocatalyst in 10 mL water/NMP (9:1 v/v), 1 M AA, and 3 wt% H_2PtCl_6

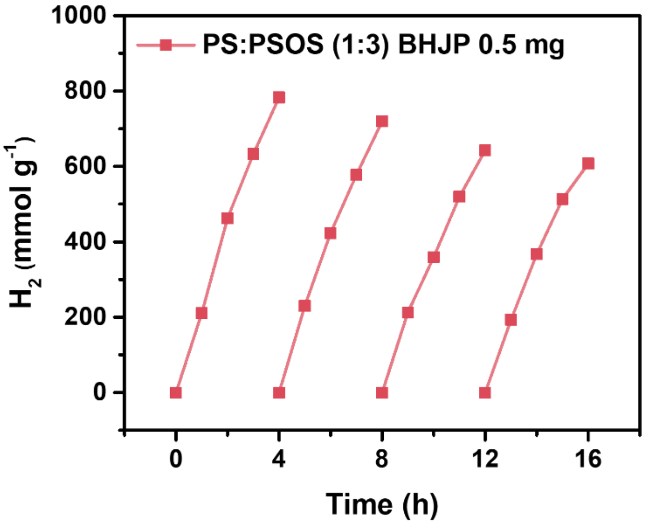

Supplementary Fig. 14 Photocatalytic cycling stability test of **PS**:**PSOS** (1:3) BHJP. Conditions: 0.5 mg photocatalyst in 10 mL water/NMP (9:1 v/v), 1 M AA, and 3 wt% H_2PtCl_6

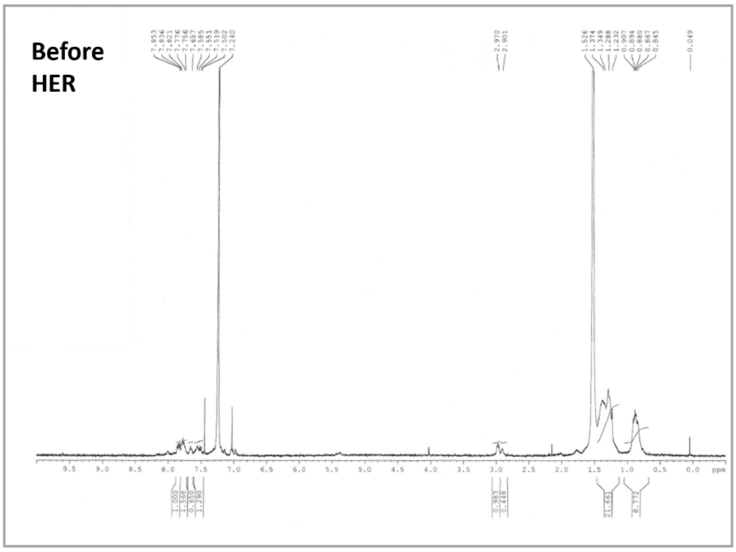

Supplementary Fig. 15 ^{1}H NMR of **PS**: **PSOS** (1:3) BHJP before HER

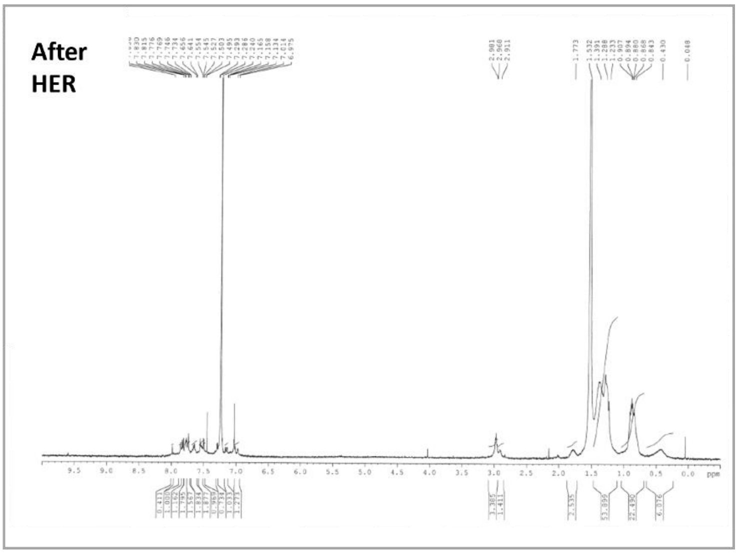

Supplementary Fig. 16 ^{1}H NMR of **PS**: **PSOS** (1:3) BHJP after HER

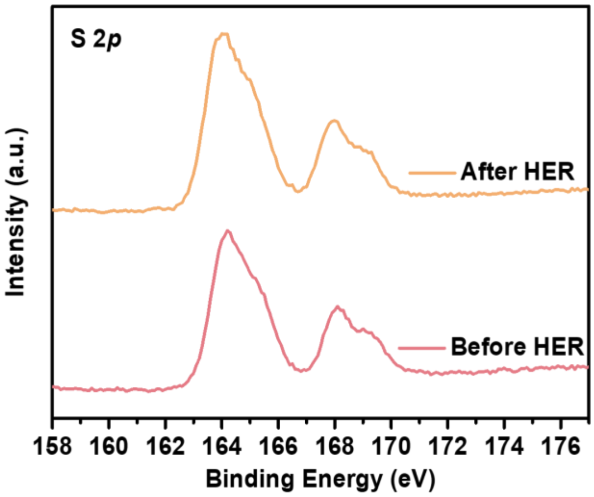

Supplementary Fig. 17 XPS of **PS**: **PSOS** (1:3) BHJP before and after HER

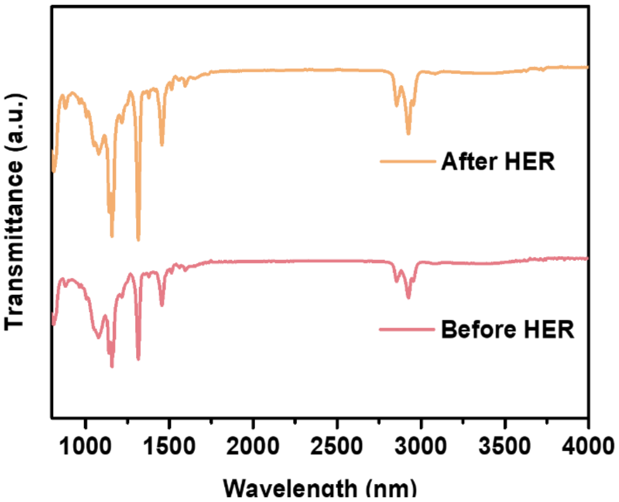

Supplementary Fig. 18 FTIR of **PS**: **PSOS** (1:3) BHJP before and after HER

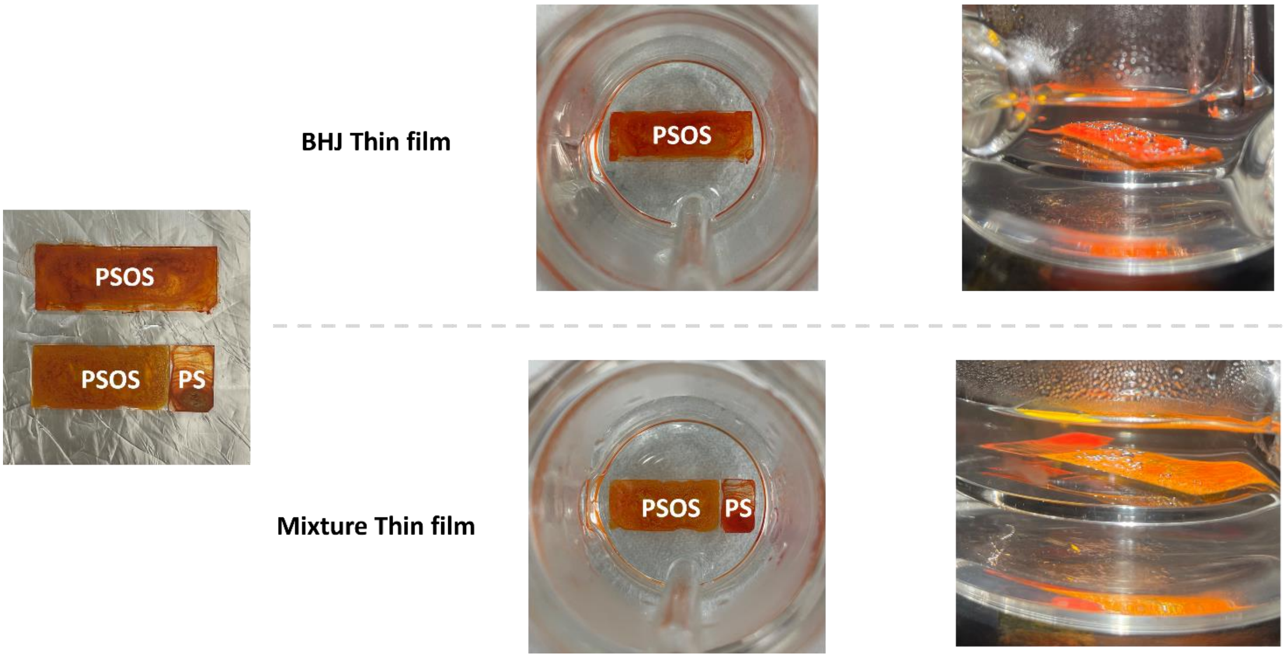

Supplementary Fig. 19 Images of **PS**: **PSOS** (1:3) BHJ thin film and mixture thin film

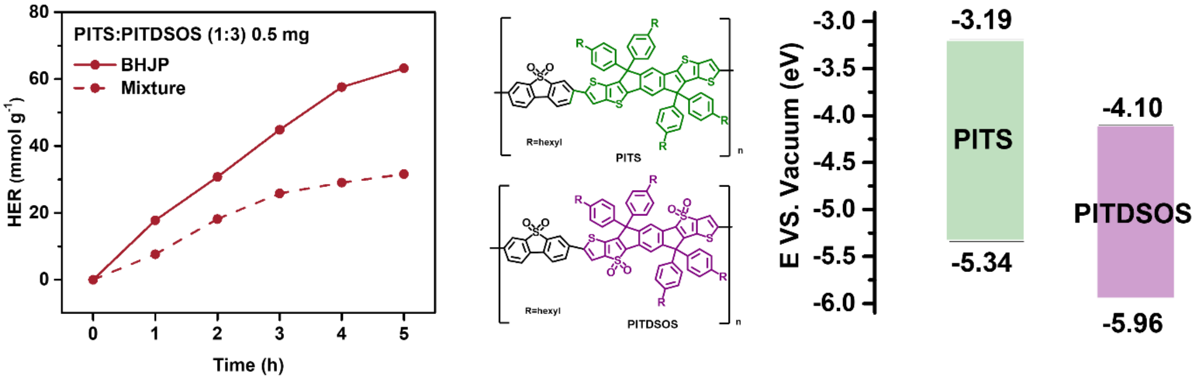

Supplementary Fig. 20 HER over time for **PITS/PITDSOS** BHJP and pristine particle mixtures. Conditions: 0.5 mg photocatalyst in 10 mL water/NMP (9:1 v/v), 1 M AA, and 3 wt% H_2PtCl_6, the molecular structures, and the energy level of the donor polymer **PITS** and the acceptor polymer **PITDSOS**

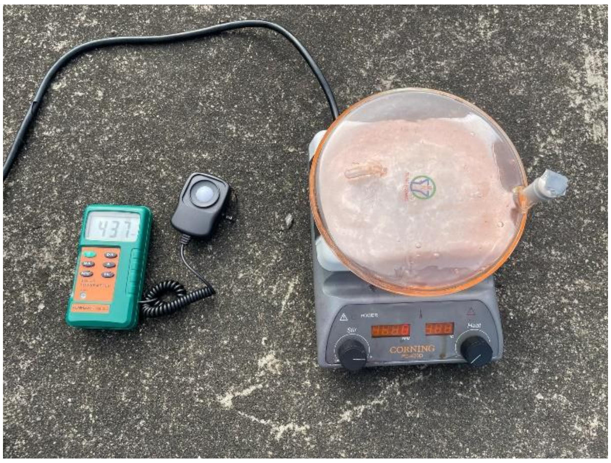

Supplementary Fig. 21 Images of a custom-designed reactor with a diameter of 20 cm and a height of 3.5 cm

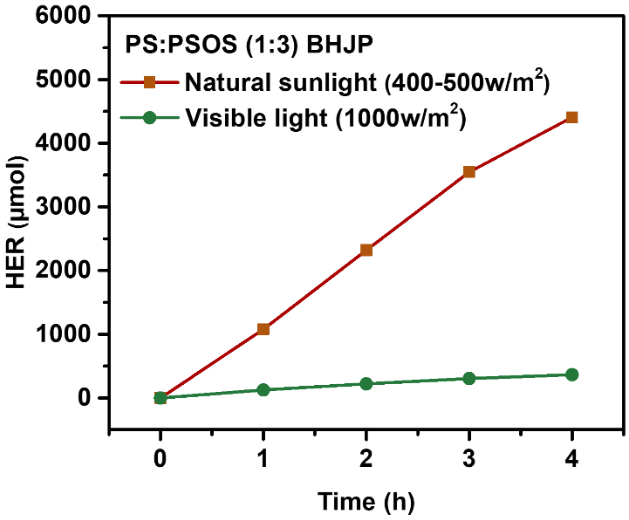

Supplementary Fig. 22 HER comparison of **PS**:**PSOS** (1:3) BHJP under natural sunlight (15 mg photocatalyst in 300 mL water/NMP, 9:1 v/v) and visible light (0.5 mg photocatalyst in 10 mL water/NMP, 9:1 v/v) with 1 M AA and 3 wt% H_2PtCl_6.

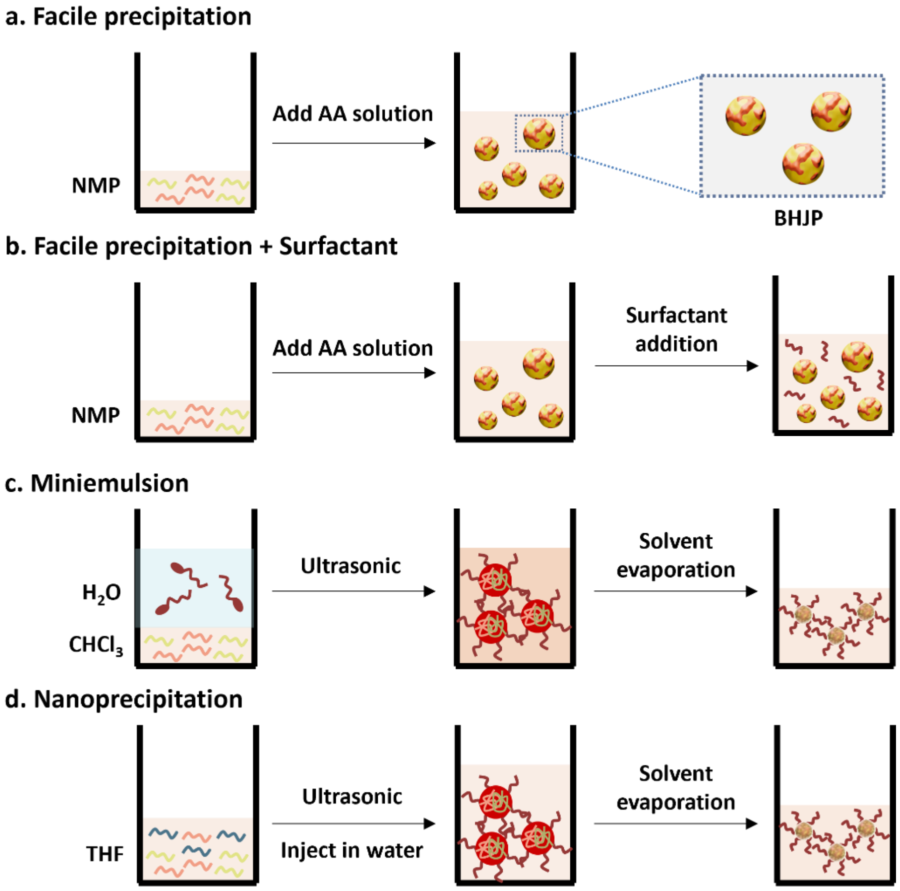

Supplementary Fig. 23 Schematic representation of the process for preparing heterojunction particles via different methods

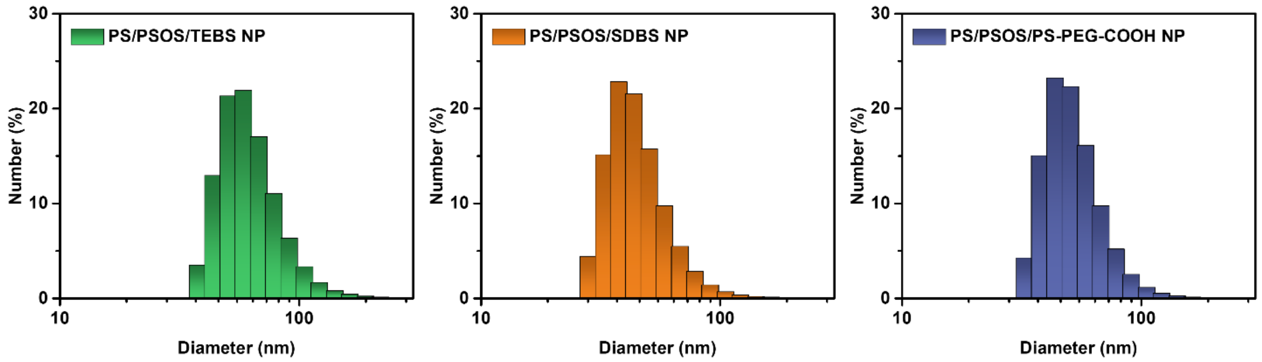

Supplementary Fig. 24 DLS of heterojunction particles prepared by mini-emulsion and nanoprecipitaion

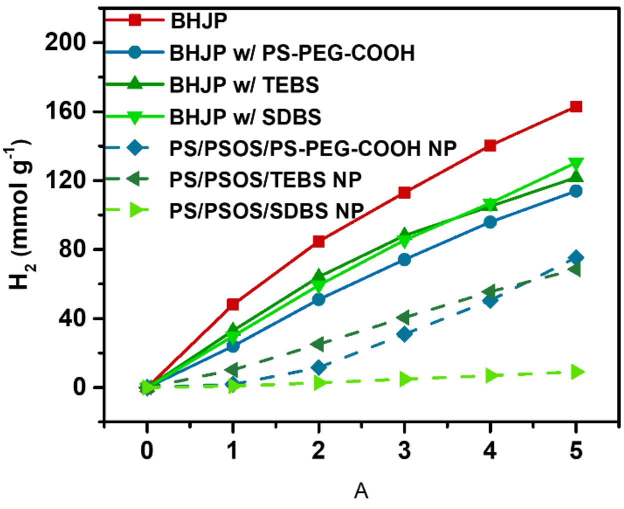

Supplementary Fig. 25 HER of **PS**/**PSOS**/Surfactant NP and **PS**/**PSOS** BHJP with additional surfactants

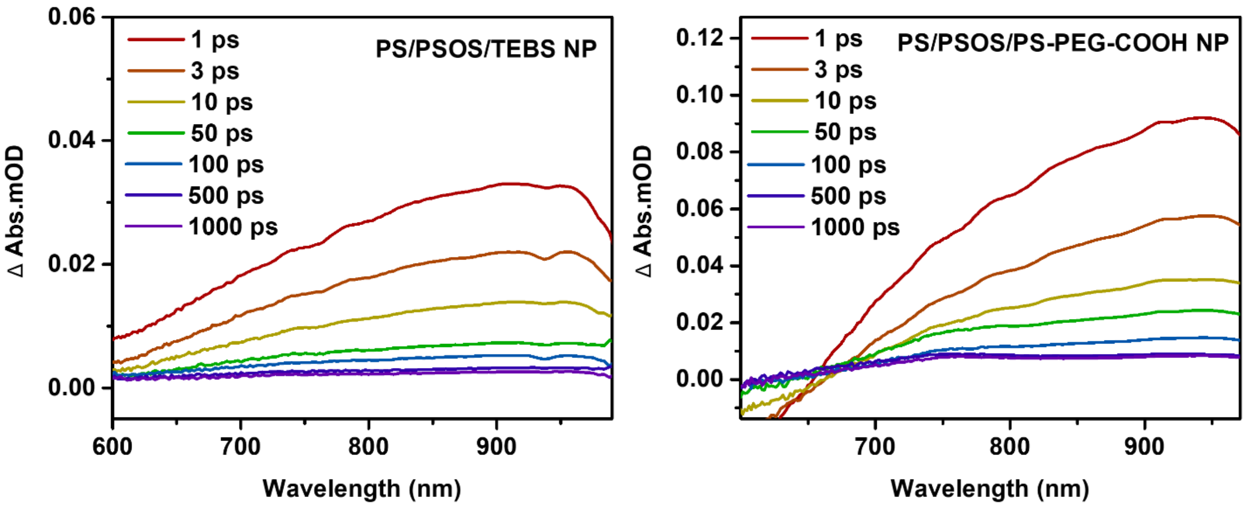

Supplementary Fig. 26 (a) TAS of **PS**/**PSOS**/**TEBS** NP prepared by mini-emulsion at various time delays following excitation at 480 nm, and (b) TAS of **PS**/**PSOS**/**PS-PEG-COOH** NP prepared by nanoprecipitation at various time delays following excitation at 480 nm

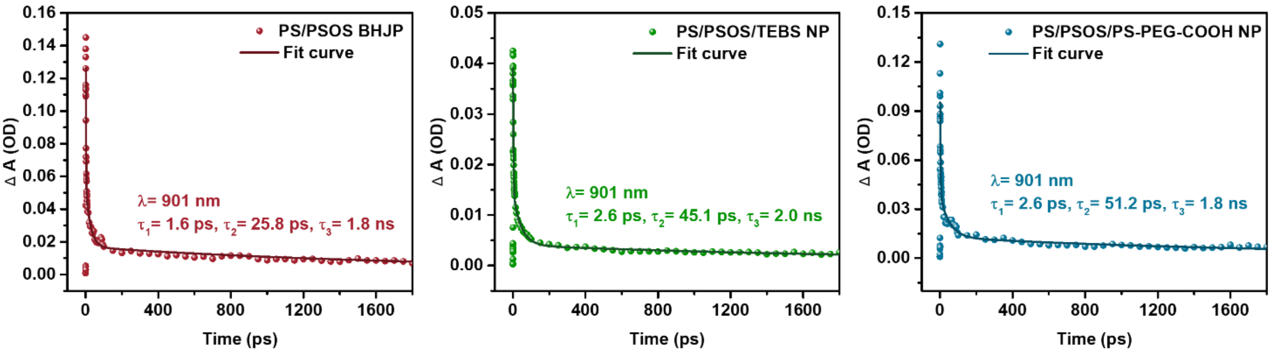

Supplementary Fig. 27 Fitting curve of the PIA signal of **PS/PSOS** heterojunction particles prepared by various methods at 901 nm

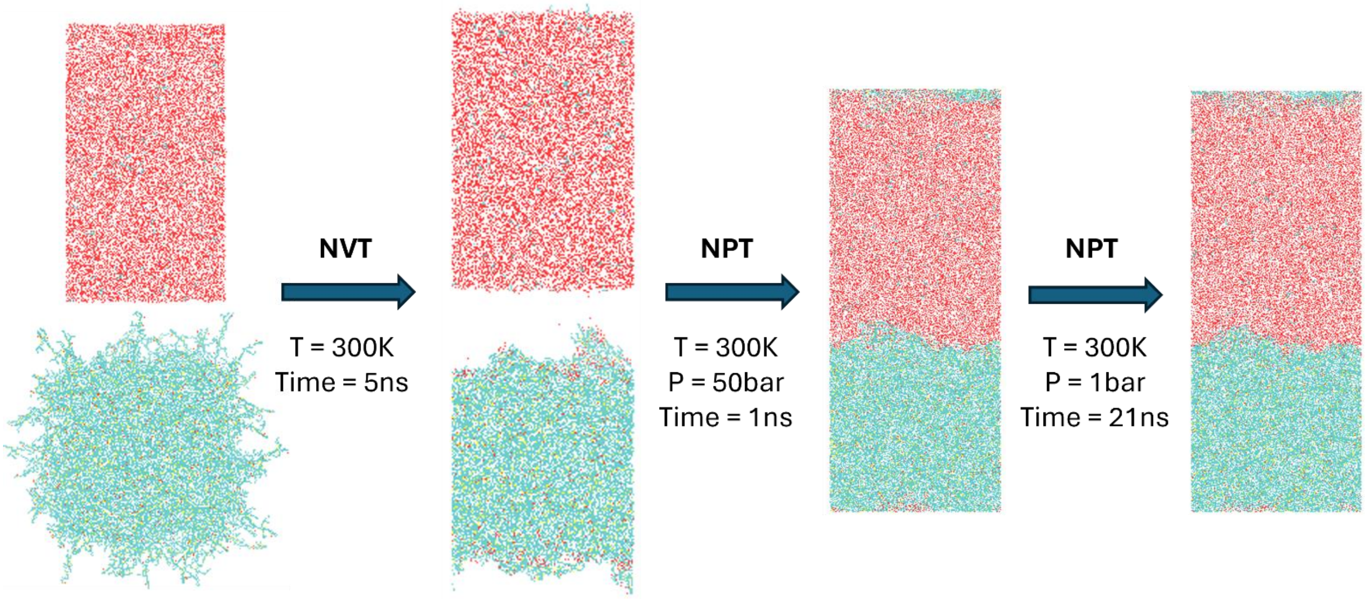

Supplementary Fig. 28 Flowchart of polymer-solution mixture simulation

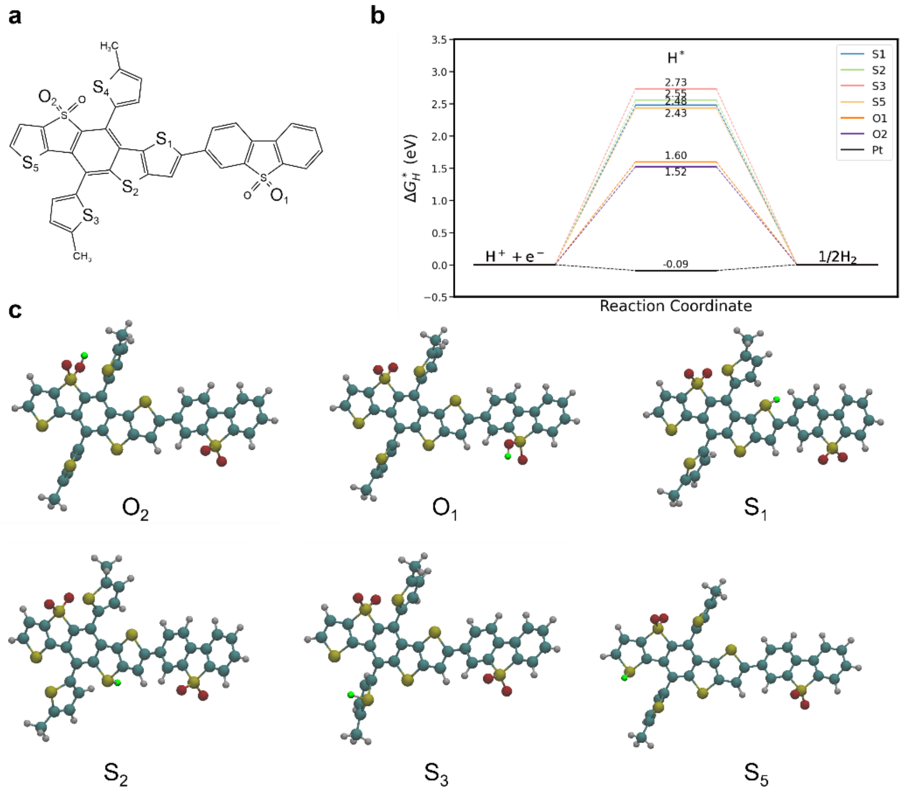

Supplementary Fig. 29 (a) The molecular structure of acceptor monomer and the tested active site (with a bigger font size) on the molecule, (b) The hydrogen adsorption free energy diagram, and (c) The optimized structures of H^* for different adsorption sites (the light green spheres represent the H atom)

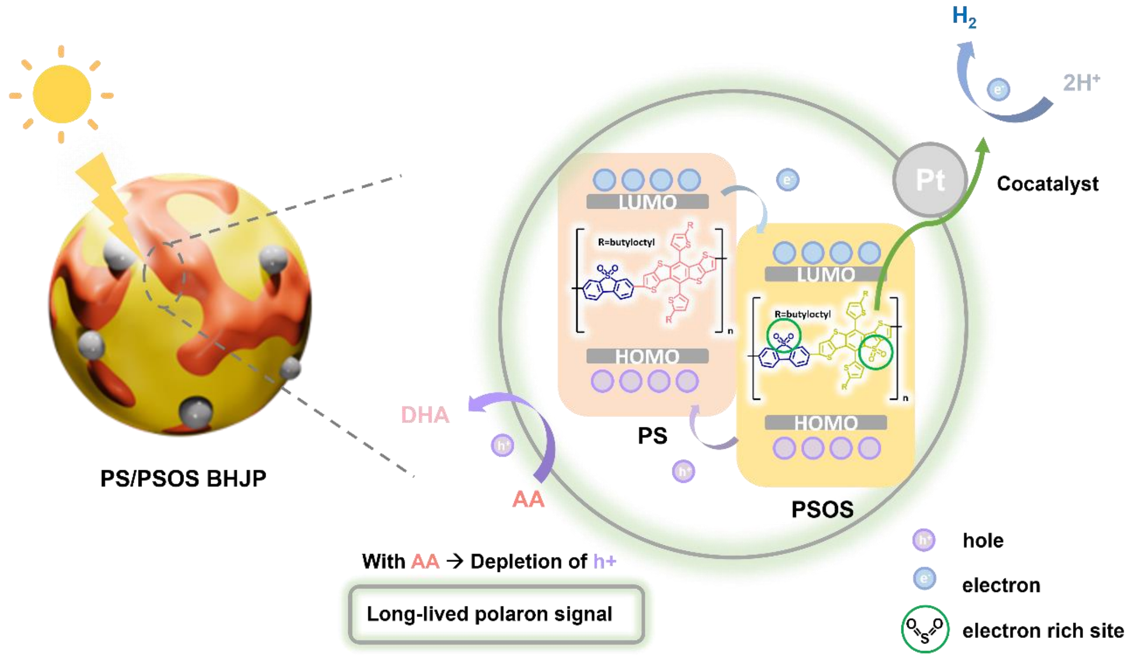

Supplementary Fig. 30 The proposed photocatalytic hydrogen evolution mechanism of **PS/PSOS** BHJP

Supplementary Table 1 Contact angles (CA), surface energy, Flory–Huggins interaction parameter (χ) and interfacial tension (γ) of the used material.

	Water CA [°]	EG CA [°]	Surface energy [mN m^{-1}]	χ_{A-B}
**PS**	97.3	79.2	20.62	$\chi_{PS-PSOS}$ =0.054K
**PSOS**	86.4	70.9	22.79	
**PM6**	88.9	68.1	27.23	χ_{PM6-Y6}=0.448K
**Y6**	99.2	67.0	34.67	

Supplementary Table 2. Potential energy (vacuum and amorphous solid), system volume, and solubility parameters from MD simulations and experimental results

Polymer	E_{vac} (kJ/mol)	E_{bulk} (kJ/mol)	Volume (nm^3)	δ_{MD} (MPa$^{1/2}$)	δ_{exp} (MPa$^{1/2}$)
**PS**	1255.15 ±59.21	279697.34 ±2093.96	5616.46 ±2.33	16.985 ±0.515	16.936
**PSOS**	1237.95 ±61.92	121071.49 ±2362.74	5716.11 ±2.08	18.016 ±0.499	17.818

Supplementary Table 3. Potential energy, volume, cohesive energy density, and energy change of mixing per unit volume for the blend polymer

	E_{bulk} (kJ/mol)	Volume (nm^3)	CED (MPa)	ΔE_{mix}	χ_{mix}
**1:1 Blend**	203622.06 ±5396.08	5673.07 ±2.26	305.38 ±17.75	1156236.21 ±21744.86	0.399 ±0.029
**1:3 Blend**	85265.22 ±5708.88	2854.44 ±2.16	314.34 ±17.83	1209212.58 ±21813.48	0.415 ±0.029

Supplementary Table 4. The fitted parameters with a mass fractal model for the SAXS data (**Fig. S4**) of the sample solutions of **PS/PSOS** prepared in different mixing ratios. The fitting parameters are D_f, r_d, and ξ, for the fractal dimension, radius of polymer particles, and cutoff length, respectively; the radius of gyration R_g is derived from $R_g = [D_f(D_f+1)/2]^{1/2}\xi$.

**Original SAXS data fitting**	**PS:PSOS (3:1)**	**PS:PSOS (1:1)**	**PS:PSOS (1:3)**
**D_f**	2.86±0.01	2.74±0.01	2.74±0.01
**r_d (Å)**	62.6±3.0	62.6±1.0	62.6±0.1
ξ **(Å)**	857±19	954±11	1061±5
R_g	2013	2159	2401

Supplementary Table 5. The fitted parameters for the repeated SAXS data (**Fig. S5**) of similar sample solutions of **PS/PSOS** prepared in different mixing ratios, using the same mass fractal model, with the fitting parameters D_f, r_d, and ξ, for the fractal dimension, radius of the primary particles, and cutoff length of the fractal clusters, respectively.

**Fitting for repeated SAXS data**	**PS:PSOS (3:1)**	**PS:PSOS (1:1)**	**PS:PSOS (1:3)**
**D_f**	2.86±0.01	2.84±0.01	2.85±0.01
**r_d (Å)**	62.6±6.4	62.6±1.1	62.6±0.8
ξ **(Å)**	857±28	954±12	1061±14
R_g	2013	2228	2485

Supplementary Table 6 Comparison of the HERs and AQYs of various photocatalysts under solution state photocatalytic systems in the literature.

Polymer catalysts	Amount of catalyst (mg)	HER (mmol g^{-1} h^{-1})	HER (μmol h^{-1})	AQY (%)	Reference
**Heterojunction nanoparticles prepared via mini-emulsion method**					
PTB7-Th/EH-IDTBR	2	64.4	128.8	2.0 @ 400 nm<br>2.3 @ 500 nm	Nat. Mater. **2020**, *19*, 559
PM6:PCBM 2:8	1	73.7	73.7	8.7 @ 400 nm<br>8.2 @ 470 nm<br>7.7 @ 560 nm	Nat. Energy. **2022**, *7*, 340
gIDTBT/oIDTBR	1	18.5	18.5	5.3 @ 400 nm<br>1.0 @ 440 nm	Adv. Mater. **2022**, *34*, 2105007
PM6:ITIC	1.5	112	168	1.3 @ 600 nm	ACS Catal. **2023**, *13*, 12730−12736
**Heterojunction nanoparticles prepared via nanoprecipitation method**					
PCDTBT:PC_{60}BM	0.3	105.2	31.56	3.16 @ 515 nm<br>3.02 @ 595 nm	Chem. Commun., **2020**, *56*, 6790
D1/D2/ITIC	0.062	60.8	3.77	2.2 @ 450 nm<br>4.6 @ 500 nm<br>6.5 @ 550 nm	J. Am. Chem. Soc. **2021**, *143*, 2875
**Polymer photocatalysts**					
30%PEG@BT-COF	10	11.14	111.4	11.2 @ 420 nm<br>9.9 @ 500 nm<br>8.9 @ 550 nm	Nat. Commun. **2021**, *12*, 3934
ZnCoP-F CP	30	2.76	83	6.92 @ 400 nm<br>5.19 @ 450 nm<br>5.50 @ 500 nm<br>5.78 @ 550 nm	Adv. Funct. Mater. **2021**, *31*, 2009819
2DSP-1	5	0.6	3	0.58 @ 475 nm	Angew. Chem. **2023**,*135*, e202302274
T1-75	5	6.28	31.4	3.2 @ 450 nm<br>6.4 @ 500 nm<br>1.8 @ 550 nm	Adv. Mater. **2023**, 2300037
COF-JLU35	5	70.8	354	1.02 @ 420 nm<br>2.57 @ 460 nm<br>2.55 @ 550 nm	J. Am. Chem. Soc. **2023**, *145*, 8364

**BHJP prepared via facile precipitation method**					
**PS: PSOS (1:3) BHJP**	**0.5/ 1/ 2**	**251.2/ 170.9/ 92.5**	**125.6/ 170.9/ 185**	**10.6 @ 420 nm 10.8 @ 460 nm 26.2 @ 500 nm 10.5 @ 550 nm**	**This work**

Supplementary Table 7 The MOC evaluation, excluding the polymers, of 1mg heterojunction particles prepared via facile precipitation and subsequent photocatalytic hydrogen production experiments.

Reagent	Quantity	Unit	Cost（$）
1-Methyl-2-pyrrolidone	1 mL	21.59/500 mL	0.043
Ascorbic acid	0.35 g	50.06/500 g	0.035
Hexachloroplatinic acid	0.03 mg	125.21/1 g	0.0038
Potassium hydroxide	0.067 g	42.24/1 kg	0.0028
Total cost			0.0846

Supplementary Table 8 The MOC evaluation, excluding the polymers, of 1mg heterojunction particles prepared via miniemulsion and subsequent photocatalytic hydrogen production experiments.

Reagent	Quantity	Unit	Cost（$）
Chloroform	1 mL	156.52 (20 L)	0.0078
Ascorbic acid	0.35 g	50.06/500 g	0.035
Hexachloroplatinic acid	0.03 mg	125.21/1 g	0.0038
Potassium hydroxide	0.067 g	42.24/1 kg	0.0028
TEBS/SDBS	50 mg	428.83(1g)/46.88(5 g)	21.44/0.4688
Total cost			21.489/0.5676

Supplementary Table 9 The MOC evaluation, excluding the polymers, of 1mg heterojunction particles prepared via nanoprecipitation and subsequent photocatalytic hydrogen production experiments.

Reagent	Quantity	Unit	Cost（$）
Tetrahydrofuran	6 mL	87.50 (4 L)	0.0875
Ascorbic acid	0.35 g	50.06 (500 g)	0.035
Hexachloroplatinic acid	0.03 mg	125.21 (1 g)	0.0038
Potassium hydroxide	0.067 g	42.24 (1 kg)	0.0028
PS-PEG-COOH	1 mg	2,097.06 (5g)	0.4194
Total cost			0.5485

Supplementary Table 10. Number of molecules in solutions with different SDBS concentrations.

	Blank	Low conc.	High conc.
H_2O	134000	134000	134000
AA	510	510	510
SDBS	0	52	510

References

1. Lin, W. C.; Chang, C. L.; Shih, C. H.; Lin, W. C.; Yu Lai, Z.; Chang, J. W.; Ting, L. Y.; Huang, T. F.; Sun, Y. E.; Huang, H. Y., Sulfide Oxidation on Ladder-Type Heteroarenes to Construct All-Acceptor Copolymers for Visible-Light-Driven Hydrogen Evolution. *Small* 2023, 2302682.
2. Guo, Q.; Lin, J.; Liu, H.; Dong, X.; Guo, X.; Ye, L.; Ma, Z.; Tang, Z.; Ade, H.; Zhang, M., Asymmetrically noncovalently fused-ring acceptor for high-efficiency organic solar cells with reduced voltage loss and excellent thermal stability. *Nano Energy* 2020, *74*, 104861.
3. Abraham, M. J.; Murtola, T.; Schulz, R.; Páll, S.; Smith, J. C.; Hess, B.; Lindahl, E., GROMACS: High performance molecular simulations through multi-level parallelism from laptops to supercomputers. *SoftwareX* 2015, *1*, 19-25.
4. Cole, D. J.; Vilseck, J. Z.; Tirado-Rives, J.; Payne, M. C.; Jorgensen, W. L., Biomolecular force field parameterization via atoms-in-molecule electron density partitioning. *J. Chem. Theory Comput.* 2016, *12* (5), 2312-2323.
5. Manz, T. A.; Limas, N. G., Introducing DDEC6 atomic population analysis: part 1. Charge partitioning theory and methodology. *RSC Adv.* 2016, *6* (53), 47771-47801.
6. Tkatchenko, A.; Scheffler, M., Accurate Molecular Van Der Waals Interactions from Ground-State Electron Density<? format?> and Free-Atom Reference Data. *Phys. Rev. Lett.* 2009, *102* (7), 073005.
7. Poelking, C.; Cho, E.; Malafeev, A.; Ivanov, V.; Kremer, K.; Risko, C.; Brédas, J.-L.; Andrienko, D., Characterization of charge-carrier transport in semicrystalline polymers: electronic couplings, site energies, and charge-carrier dynamics in poly (bithiophene-alt-thienothiophene)[PBTTT]. *J. Phys. Chem. C* 2013, *117* (4), 1633-1640.
8. Martínez, L.; Andrade, R.; Birgin, E. G.; Martínez, J. M., PACKMOL: A package for building initial configurations for molecular dynamics simulations. *J. Comput. Chem.* 2009, *30* (13), 2157-2164.
9. Luo, Z.; Jiang, J., Molecular dynamics and dissipative particle dynamics simulations for the miscibility of poly (ethylene oxide)/poly (vinyl chloride) blends. *Polymer* 2010, *51* (1), 291-299.
10. Kouijzer, S.; Michels, J. J.; van den Berg, M.; Gevaerts, V. S.; Turbiez, M.; Wienk, M. M.; Janssen, R. A., Predicting morphologies of solution processed polymer: fullerene blends. *J. Am. Chem. Soc.* 2013, *135* (32), 12057-12067.
11. Shih, O.; Liao, K.-F.; Yeh, Y.-Q.; Su, C.-J.; Wang, C.-A.; Chang, J.-W.; Wu, W.-R.; Liang, C.-C.; Lin, C.-Y.; Lee, T.-H., Performance of the new biological small-and wide-angle X-ray scattering beamline 13A at the Taiwan Photon Source. *J. Appl. Crystallogr.* 2022, *55* (2), 340-352.
12. Teixeira, J., Small-angle scattering by fractal systems. *J. Appl. Crystallogr.* 1988, *21* (6), 781-785.
13. Woods, D. J.; Hillman, S. A.; Pearce, D.; Wilbraham, L.; Flagg, L. Q.; Duffy, W.; McCulloch, I.; Durrant, J. R.; Guilbert, A. A.; Zwijnenburg, M. A., Side-chain tuning in conjugated polymer photocatalysts for improved hydrogen production from water. *Energy Environ. Sci.* 2020, *13*, 1843.
